# Image‐Based Biological Heart Age Estimation Reveals Differential Aging Patterns Across Cardiac Chambers

**DOI:** 10.1002/jmri.28675

**Published:** 2023-03-16

**Authors:** Ahmed M. Salih, Esmeralda Ruiz Pujadas, Víctor M. Campello, Celeste McCracken, Nicholas C. Harvey, Stefan Neubauer, Karim Lekadir, Thomas E. Nichols, Steffen E. Petersen, Zahra Raisi‐Estabragh

**Affiliations:** ^1^ William Harvey Research Institute, NIHR Barts Biomedical Research Centre Queen Mary University of London London UK; ^2^ Departament de Matemàtiques i Informàtica Universitat de Barcelona, Artificial Intelligence in Medicine Lab (BCN‐AIM) Barcelona Spain; ^3^ Division of Cardiovascular Medicine, Radcliffe Department of Medicine, University of Oxford, National Institute for Health Research Oxford Biomedical Research Centre Oxford University Hospitals NHS Foundation Trust Oxford UK; ^4^ MRC Lifecourse Epidemiology Centre University of Southampton Southampton UK; ^5^ NIHR Southampton Biomedical Research Centre University of Southampton and University Hospital Southampton NHS Foundation Trust Southampton UK; ^6^ Wellcome Centre for Integrative Neuroimaging, FMRIB, Nuffield Department of Clinical Neurosciences University of Oxford Oxford UK; ^7^ Big Data Institute, Li Ka Shing Centre for Health Information and Discovery, Nuffield Department of Population Health University of Oxford Oxford UK; ^8^ Barts Heart Centre, St Bartholomew's Hospital, Barts Health NHS Trust, West Smithfield London UK; ^9^ Health Data Research UK London UK; ^10^ Alan Turing Institute London UK

**Keywords:** aging, cardiac imaging, cardiac health, radiomics

## Abstract

**Background:**

Biological heart age estimation can provide insights into cardiac aging. However, existing studies do not consider differential aging across cardiac regions.

**Purpose:**

To estimate biological age of the left ventricle (LV), right ventricle (RV), myocardium, left atrium, and right atrium using magnetic resonance imaging radiomics phenotypes and to investigate determinants of aging by cardiac region.

**Study type:**

Cross‐sectional.

**Population:**

A total of 18,117 healthy UK Biobank participants including 8338 men (mean age = 64.2 ± 7.5) and 9779 women (mean age = 63.0 ± 7.4).

**Field Strength/Sequence:**

A 1.5 T/balanced steady‐state free precession.

**Assessment:**

An automated algorithm was used to segment the five cardiac regions, from which radiomic features were extracted. Bayesian ridge regression was used to estimate biological age of each cardiac region with radiomics features as predictors and chronological age as the output. The “age gap” was the difference between biological and chronological age. Linear regression was used to calculate associations of age gap from each cardiac region with socioeconomic, lifestyle, body composition, blood pressure and arterial stiffness, blood biomarkers, mental well‐being, multiorgan health, and sex hormone exposures (*n* = 49).

**Statistical Test:**

Multiple testing correction with false discovery method (threshold = 5%).

**Results:**

The largest model error was with RV and the smallest with LV age (mean absolute error in men: 5.26 vs. 4.96 years). There were 172 statistically significant age gap associations. Greater visceral adiposity was the strongest correlate of larger age gaps, for example, myocardial age gap in women (Beta = 0.85, *P* = 1.69 × 10^−26^). Poor mental health associated with large age gaps, for example, “disinterested” episodes and myocardial age gap in men (Beta = 0.25, *P* = 0.001), as did a history of dental problems (eg LV in men Beta = 0.19, *P* = 0.02). Higher bone mineral density was the strongest associate of smaller age gaps, for example, myocardial age gap in men (Beta = −1.52, *P* = 7.44 × 10^−6^).

**Data Conclusion:**

This work demonstrates image‐based heart age estimation as a novel method for understanding cardiac aging.

**Evidence Level:**

1.

**Technical Efficacy:**

Stage 1.

Epidemiologic trends indicate aging global populations and increasing burden from diseases of older age.^1^ Cardiovascular diseases (CVDs) are the most common cause of disability and premature death worldwide and occur more commonly in older individuals.^2^ Optimizing healthy cardiac aging is a global public health priority.^3^

Cardiac imaging may capture distinct age‐related cardiac alterations. Magnetic resonance imaging (MRI) is the reference modality for cardiac chamber quantification and can provide evaluation of myocardial tissue character.^4^

MRI derived phenotypes (IDPs) permit noninvasive characterization of cardiovascular health and detection of preclinical organ‐level remodeling.^5^ Alteration of MRI phenotypes reflects exposure to specific cardiovascular stressors, which may differentially impact individual cardiac chambers. For instance, chronic pulmonary disorders are known to preferentially impact right atrial and right ventricular phenotypes^6^; while hypertension‐related remodeling primarily affects the left heart.^7^ Thus, the exposure profile of an individual can determine the pattern of aging across different heart structures. The recognition of such remodeling patterns is important, as they have different clinical and prognostic consequences. Furthermore, the pattern of remodeling associated with an exposure can provide insight into the mechanisms through which it alters cardiovascular health.

In previous reports, researchers have used deep learning methods applied to cardiovascular imaging to develop estimates of heart age.^8^, ^9^ These studies present novel approaches to evaluating heart age based on its image appearance. However, given the “black box” nature of these methods, the interpretability of the developed models is limited. Importantly, it is not possible to highlight the precise impact of an exposure on specific cardiac structures. This severely limits biological and clinical inferences from such models.

MRI radiomics analysis permits extraction of many quantitative measures of cardiac shape and myocardial character using voxel‐level data.^5^ The large number of features generated lends itself ideally to machine learning methods. A key advantage of MRI radiomics features over black box methods is the potential to produce interpretable models.

We hypothesized that heart age of individual cardiac structures may be modeled using radiomics features extracted from related regions, that is, it may be possible to describe, in a quantitative and interpretable manner, differential aging patterns across cardiac chambers. This information could in turn be used to evaluate patterns of aging across different cardiovascular structures ascribed to specific exposures.

The aim of this study was to use MRI radiomics features to estimate biological age of the left and right ventricles (LV, RV) and atria (LA, RA), and the LV myocardium. A further aim was to investigate the association of selected exposures on aging across these structures, separately in men and women.

## Materials and Methods

### Data Source and Population Characteristics

The UK Biobank comprises detailed characterization of approximately 500,000 individuals from across the United Kingdom. The participants were aged 40–69 at recruitment (2006–2010). Baseline assessment was conducted according to a published research protocol,^10^ gathering information on demographic, lifestyle and environment factors, cognitive tests, and blood sampling. The UK Biobank Imaging Study was launched in 2015 and is ongoing, aiming to perform multiorgan imaging for a 20% (*n* = 100,000) subset of the original participants. In this study, we included 29,144 participants for whom MRI data were available. We excluded 11,027 participants with history of CVD at time of imaging (Table S1 Supplemental Material). The analysis sample included 8338 men and 9779 women. The average age was 64.2 (±7.5) years for men and 63.0 (±7.4) years for women.

### Image Acquisition

Imaging was performed in dedicated UK Biobank centers using uniform staff training, equipment, and predefined acquisition protocols.^11^ MRI scans were performed using 1.5 T scanners (MAGNETOM Aera, Syngo Platform VD13A, Siemens Healthcare, Erlangen, Germany). Cardiac structure and function were assessed using standard long‐axis slices (vertical long axis, horizontal long axis, and left ventricular outflow tract) and a short‐axis stack covering the ventricles from base to apex. All cine images were acquired with a balanced steady‐state free precession sequence. The imaging protocol parameters were set to as slice thickness (6.0), matrix size (208 × 187), voxel size (1.8 × 1.8 × 6.0), TR (msec) (2.7), TE (msec) (1.16), and acquired temporal resolution (msec) (32.64). Further details of pulse sequence parameters have been previously published.^11^

### Image Segmentation

We computed radiomics features from the voxels identified by the atrial contours from long axis and the RV, LV, and LV myocardium from short‐axis images, in end‐systole and end‐diastole. Automated segmentation of the ventricular and myocardial regions was performed using a previously developed pipeline, trained on a large expert annotated manual segmentation dataset. End‐diastolic was considered as the first phase of the acquisition. Experts determined the end‐systolic phase visually by which the LV intracavity blood pool is in its smallest size at the mid‐ventricular level.^12^

To define the atrial contours from the long‐axis images, an automatic segmentation model based on a traditional U‐Net architecture was implemented. Ground truth manually annotated datasets (*n* = 764) were used for model fitting.^12^ Data augmentation techniques were used to introduce more variability in the overall structure and appearance of images and to improve the generalizability of the model. These included small rotations of the image, random bias field perturbations, random contrast adjustments, and random intensity histogram shifting. The model was trained for 100 epochs with a batch size of 16 on 256 × 256 images using the Adam optimizer with a learning rate of 0.0001 and 0.9 and 0.999 first and second moments, respectively. Binary cross entropy was used as loss function. The resulting model was used to generate automatic delineations for the rest of the studies considered in this work. Two postprocessing steps were used to ensure smooth contours: an algorithm to fill potential holes in the final mask and a selection of the largest connected component predicted for each region of interest (ROI). A fully automated quality‐controlled image analysis pipeline, previously developed and validated in a large subset of the UK Biobank,^12^, ^13^ was applied to short‐axis images to define the LV, RV, and myocardial contours.

For each study, the RV, LV, and myocardial contours were automatically defined and exported in a single xml file. We developed an in‐house software in Python (version 3.7.9) to convert the contours into binary masks, which we have made publicly available.^14^ This software builds a polygon from the contour points in the coordinate space to form the mask, given the xml file and the corresponding MR DICOM images. The area bounded by the contour in every slice was filled with ones using the fillpoly function from the OpenCV^15^ library, resulting in the binary ROI. This process was repeated for all delineated contours. For the atrial contours, the deep learning method was designed to automatically return a binary mask without the need for any intermediate steps.

### Feature Extraction

The open‐source PyRadiomics platform (version 2.2.0.) was used to extract Radiomics features given the contours and the corresponding images. For intensity‐based and texture features, brightness harmonization was achieved by histogram standardization, and gray values were discretized with a bin width of 25 (units). For each frame, we computed 13 shape, 18 first‐order, and 75 texture features. In the long axis, one 3D shape feature, “flatness,” was discarded in outlier removal checks. The texture features were extracted using five different matrices: gray‐level co‐occurrence matrix (24 features), gray‐level run‐length matrix (16 features), gray‐level size‐zone matrix (16 features), neighboring gray tone difference matrix (5 features), and gray‐level dependence matrix (14 features). In all, we computed a total of 210 radiomics features for each ROI (shape *n* = 24, first order *n* = 36, texture *n* = 150). The full list of the radiomic features extracted is displayed in Table S2 Supplemental Material. Further background information to radiomics can be found in dedicated review articles.^5^, ^16^, ^17^, ^18^

### Feature Selection

All the following steps were implemented using Python 3.8.10 and Scikit‐learn 1.0.2. A total of 1050 radiomics features were available (210 from each of 5 ROIs [LV, RV, LA, RA, myocardium]). We built individual models for each ROI, separately for men and women, resulting in a total of 10 models. Model development methods were uniform across all 10 models. First, we applied recursive feature elimination with cross‐validation (RFECV) to choose the optimal number of features (among the 210 per ROI) using Bayesian ridge regression^19^ as the model (10‐fold), and with chronological age set as the dependent variable. Thereafter, we applied Cook's Distance^20^ method to detect and remove any outliers. A data point was considered an outlier by Cook's distance if its value was larger than three times the mean of all the data points (Table S3 Supplemental Material).

### Model Building

**FIGURE 1:**
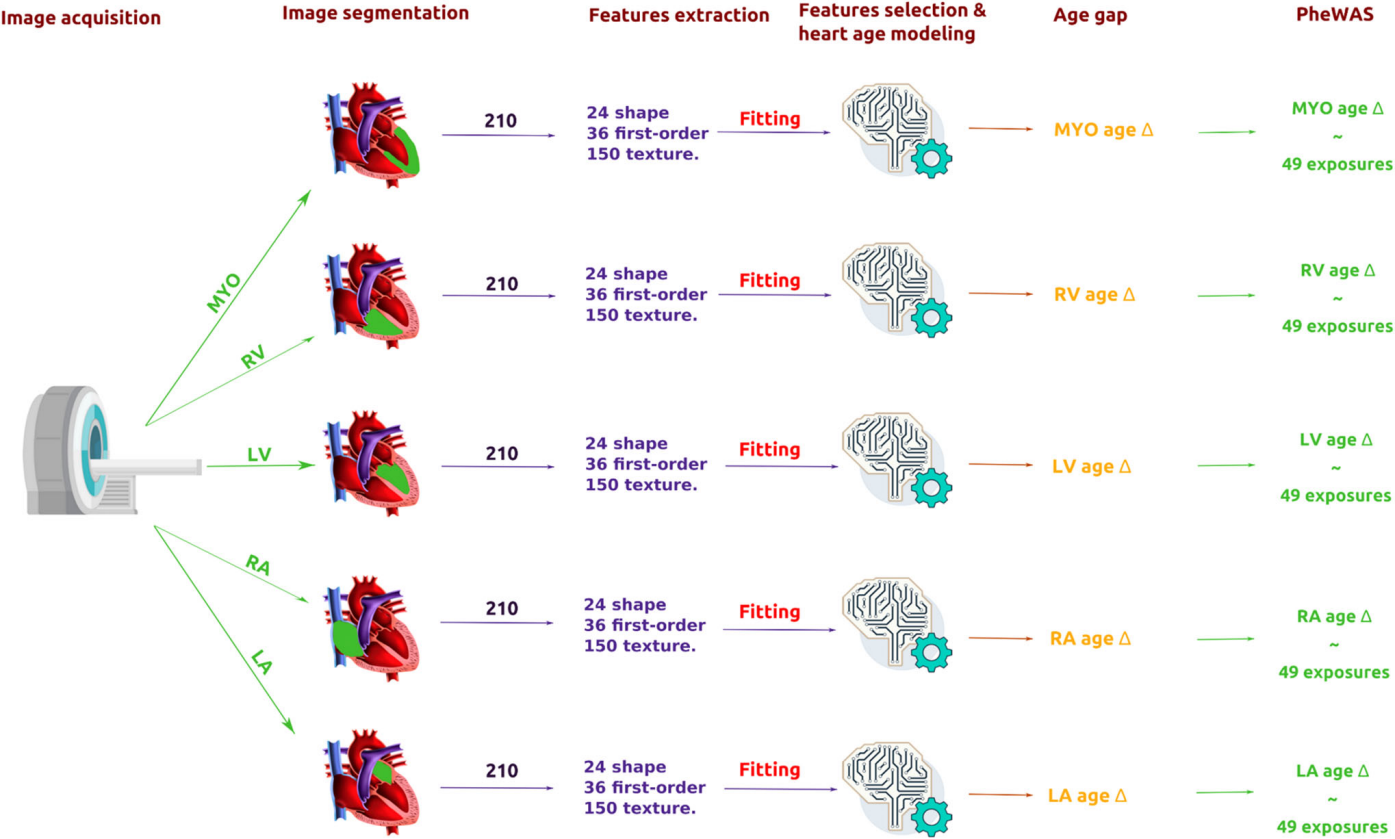
Conceptual overview of the steps used to estimate heart age for each cardiac region and perform PheWAS. These steps were performed separately for women and men. LA = left atrium; LV = left ventricle; MYO = myocardium; PheWAS = phenome wide association study; RA = right atrium; RV = right ventricle. The full list of exposures is presented in Table S4 in the Supplemental Material.

Figure 1 explains the overall of the study and modeling. Height and weight were considered as confounds and regressed out from the features using a linear regression model where the confounds are the independent variables and each feature is the dependent variable. Thereafter, the features were normalized to have zero mean and unit variance. Bayesian ridge regression was used to estimate the age of each ROI. The “age gap” values were calculated by subtracting the actual age from the predicted age for each cardiac structure (or ROI). We examined the association of age gap metrics with lifestyle and health exposures.

### Explainability

To aid interpretability of our models, we identified the most informative features driving the model output using the SHapley Additive exPlanations (SHAP) method. SHAP calculates a value for each radiomics feature representing the contribution of that feature to the model output. The output of SHAP is a list of the most informative features in the model in descending order. The list is based on the SHAP value for each feature in the model, which quantifies the impact (magnitude, direction) of the feature on the model output.

### Associations of Age Gap With Selected Exposures

We considered associations between age gap from each structural region and a selection of key exposures selected based on biological knowledge of their associations with cardiovascular health. We considered 49 exposures (Table S4 Supplemental Material), including socioeconomic factors (*n* = 5), lifestyle factors (*n* = 6), obesity and body composition metrics (*n* = 9), blood pressure and arterial stiffness (*n* = 4), blood biomarkers (*n* = 7), mental well‐being (*n* = 6), multiorgan health indicators (*n* = 10), and sex hormones (*n* = 2). The following quality control steps were performed on the exposures before investigating the association with age gap. Levels within categorical variables were re‐ordered to align higher scores with healthier exposure levels (applies to educational level, health satisfaction, financial satisfaction). Blood pressure and resting heart rate were limited to biologically plausible ranges: systolic blood pressure: >60 mmHg and <200 mmHg; diastolic blood pressure: >40 mmHg and <120 mmHg, resting heart rate: >40 bpm and <140 bpm. Blood biochemistry parameters were restricted to values within the manufacturer's analytical range.^21^

### Statistical Analysis

Model performance was assessed using mean absolute error (MAE) and prediction coefficient of determination (*R* ^2^). MAE is the difference between the predicted value (estimated heart age) and the actual value (actual age). Prediction *R* ^2^ measures how much of the variation in the outcome (predicted heart age) is explained by the input data (radiomics features) and is calculated as: Prediction *R* ^2^ = 1‐ Prediction mean squared error/total sum of squares. We applied a regression to the mean correction to remove dependency of heart age gap (delta) on age.^22^

Linear regression was used to examine associations of heart age gap from each ROI with each exposure. Models were adjusted for height, weight, and age. We report beta coefficients and 95% confidence intervals (CI) relating to the age gap value associated with each exposure—which indicates difference in cardiac age (for each anatomic region) for each unit increase in the exposure. A positive beta value indicates direction of association toward a more positive age gap—that is, greater cardiac age than actual age (likely adverse exposure). We corrected for multiple testing using the false discovery rate method (threshold *P* < 0.05).

## Results

### Baseline Characteristics

Compared to women, men had poorer cardiometabolic profile, with greater obesity, poorer glycemic control, and higher blood pressure and arterial stiffness. Women had, on average, greater levels of deprivation, lower educational level, and lived in lower income households. Women also scored higher on all indicators of poorer mental well‐being. More details of baseline population characteristics are explained in Table 1.

**TABLE 1:** Baseline Population Characteristics

	Men	Women
*n*	8338	9779
Age (years)	64.17 (±7.50)	63.04 (±7.36)
Cardiac MRI metric		
Left ventricular end‐diastolic volume index	82.83 (±14.32)	73.50 (±10.91)
Left ventricular mass index	50.94 (±7.7)	40.66 (±5.81)
Left ventricular ejection fraction	58.02 (±5.99)	61.02 (±5.59)
Right ventricular end‐diastolic volume index	89.07 (±15.16)	76.13 (±11.89)
Right ventricular ejection fraction	55.28 (±5.78)	59.25 (±5.63)
Socioeconomic factors		
Townsend score	−2.70 [−3.93–0.62]	−2.55 [−3.85–0.47]
Education level		
None	1 [6.04%]	1 [5.84%]
Secondary education	2 [4.00%]	2 [3.92%]
High school diploma	3 [16.87%]	3 [21.22%]
Vocational diploma	4 [7.71%]	4 [3.24%]
Other professional qualifications, for example, nursing, teaching	5 [4.13%]	5 [6.20%]
A levels/AS levels or equivalent	6 [11.85%]	6 [14.31%]
College or University degree	7 [49.41%]	7 [45.28%]
Number of vehicles in household		
1, None	1 [3.24%]	1 [4.10%]
2, One	2 [34.35%]	2 [39.39%]
3, Two	3 [47.68%]	3 [43.47%]
4, Three	4 [11.49%]	4 [9.95%]
5, Four or more	5 [3.24%]	5 [3.09%]
Average total household income before tax		
1, Less than £18,000	1 [8.92%]	1 [13.55%]
2, 18,000–30,999	2 [20.30%]	2 [24.86%]
3, 31,000–51,999	3 [31.63%]	3 [30.03%]
4, 52,000–100,000	4 [31.15%]	4 [26.00%]
5, Greater than 100,000	5 [8.00%]	5 [5.55%]
Number of people in household	2.54 [1.16]	2.48 [1.15]
Lifestyle factors		
Time spent watching television (hours/day)	2 [1–3]	2 [1–3]
Oily fish intake		
0 = Never	0 [9.22%]	0 [9.52%]
1 = Less than once a week	1 [36.14%]	1 [33.52%]
2 = Once a week	2 [37.61%]	2 [40.03%]
3 = 2–4 times a week	3 [15.99%]	3 [16.38%]
4 = 5–6 times a week	4 [0.81%]	4 [0.49%]
5 = Once or more daily	5 [0.22%]	5 [0.05%]
Beef intake		
0 = Never	0 [7.38%]	0 [13.54%]
1 = Less than once a week	1 [47.06%]	1 [45.55%]
2 = Once a week	2 [33.88%]	2 [29.58%]
3 = 2–4 times a week	3 [11.48%]	3 [11.25%]
4 = 5–6 times a week	4 [0.16%]	4 [0.06%]
5 = Once or more daily	5 [0.04%]	5 [0.01%]
Pork intake		
0 = Never	0 [11.67%]	0 [19.49%]
1 = Less than once a week	1 [60.84%]	1 [59.35%]
2 = Once a week	2 [23.65%]	2 [18.74%]
3 = 2–4 times a week	3 [3.71%]	3 [2.38%]
4 = 5–6 times a week	4 [0.10%]	4 [0.04%]
5 = Once or more daily	5 [0.02%]	5 [0%]
Number of days/week of moderate physical activity 10+ minutes (days/week)	3.33 [2.24]	3.50 [2.28]
Smoking status		
0, Never	0 [56.05%]	0 [63.87%]
1, Previous	1 [36.81%]	1 [31.19%]
2, Current	2 [7.14%]	2 [4.94%]
Obesity and body composition metrics		
Visceral adipose tissue volume (liters)	4.77 [3.33–6.49]	2.37 [1.51–3.57]
Abdominal subcutaneous adipose tissue volume (liters)	5.53 [4.29–7.16]	7.59 [5.66–9.94]
Total trunk fat volume (liters)	10.97 [4.29]	10.71 [4.61]
Body mass index (kg/m^2^)	26.85 [24.76–29.37]	25.43 [23.09–28.85]
Whole body fat mass (kg)	21.45 [7.23]	25.74 [8.88]
Waist circumference (cm)	95.13 [10.26]	82.51 [11.13]
Liver PDFF (proton density fat fraction, %)	2.92 [1.80–6.13]	1.98 [1.33–3.81]
Total lean tissue volume (liters)	27.38 [25.77–29.87]	20.25 [18.60–22.14]
Trunk fat mass (kg)	14.02[4.54]	13.23[4.84]
Blood pressure and arterial stiffness		
Pulse rate (bpm)	67 [60–74]	68 [62–75]
Pulse wave arterial stiffness index (m/sec)	9.63 [7.72–11.83]	8.12 [6.20–10.33]
Diastolic blood pressure, automated reading (mmHg)	84.10 [9.93]	79.88 [10.28]
Systolic blood pressure, automated reading (mmHg)	141.31 [16.81]	134.19 [18.99]
Blood biomarkers		
Alanine aminotransferase (units per liter)	23.97 [18.63–32.18]	17.19 [13.62–22.47]
Gamma glutamyltransferase (units per liter)	31.90 [23.10–47.30]	20.25 [15.30–28.80]
Glycated hemoglobin (mmol/mol)	34.80 [32.40–37.30]	34.60 [32.30–37.10]
Triglycerides level (mmol/liter)	1.67 [1.18–2.38]	1.27 [0.93–1.78]
Cholesterol (mmol/liter)	5.55 [1.07]	5.86 [1.07]
LDL (mmol/liter)	3.54 [0.83]	3.62 [0.83]
HDL (mmol/liter)	1.26 [1.08–1.46]	1.58 [1.35–1.83]
Mental health		
Fed‐up feelings		
1, Yes	1 [33.78%]	1 [40.13%]
0, No	0 [66.22%]	0 [59.87%]
Nervous feelings		
1, Yes	1 [18.22%]	1 [23.98%]
0, No	0 [81.78%]	0 [76.02%]
Neuroticism score	3.52 [3.17]	4.47 [3.20]
Health satisfaction		
1, Extremely unhappy	1 [0.34%]	1 [0.74%]
2, Very unhappy	2 [1.47%]	2 [1.67%]
3, Moderately unhappy	3 [7.11%]	3 [7.94%]
4, Moderately happy	4 [48.69%]	4 [48.59%]
5, Very happy	5 [37.07%]	5 [35.93%]
6, Extremely happy	6 [5.32%]	6 [5.12%]
Financial situation satisfaction		
1, Extremely unhappy	1 [0.73%]	1 [0.90%]
2, Very unhappy	2 [1.77%]	2 [1.67%]
3, Moderately unhappy	3 [5.19%]	3 [5.59%]
4, Moderately happy	4 [38.55%]	4 [37.86%]
5, Very happy	5 [41.06%]	5 [41.84%]
6, Extremely happy	6 [12.71%]	6 [12.14%]
Ever unenthusiastic/disinterested for a whole week		
1, Yes	1 [29.89%]	1 [41.47%]
0, No	0 [70.11%]	0 [58.53%]
Multiorgan health indicators		
Number of treatments/medications taken	2 [0–3]	2 [1–4]
Overall health rating		
1, Poor	1 [1.86%]	1 [2.17%]
2, Fair	2 [17.69%]	2 [15.73%]
3, Good	3 [61.81%]	3 [63.30%]
4, Excellent	4 [18.63%]	4 [18.79%]
Fluid intelligence score	6.67 [2.14]	6.39 [1.94]
Mouth/teeth dental problems		
0, No	0 [66.58%]	0 [63.23%]
1, Yes (mouth ulcers, painful gums, bleeding gums, loose teeth, toothache, dentures)	1 [33.42%]	1 [36.77%]
Heel bone mineral density (g/m^2^)	0.58 [0.14]	0.53 [0.12]
Hand grip strength (left, kg)	40 [34–46]	24 [20–28]
Hand grip strength (right, kg)	42 [36–48]	26 [22–30]
Forced expiratory volume in 1‐second (FEV1), Best measure (liters)	3.42 [0.68]	2.50 [0.50]
Forced vital capacity (FVC, liters)	4.54 [1.06]	3.24 [0.61]
Peak expiratory flow (PEF, liters/min)	502 [427–573]	350 [300–400]
Sex hormones		
Oestradiol	202.30 [187.45–223.15]	410.7 [275.5–674.3]
Testosterone	13.12 [10.9–15.4]	1.1 [0.9–1.5]

Discrete variables are presented as number (percentage). Continuous measures are mean (±SD) if normal distribution. Continuous measures are median [25th percentile, 75th percentile] if skewed distribution. All individuals with answers: do not know (−1) and prefer not to answer (−3) were removed.

MRI = magnetic resonance imaging.

### Model Performance

We present model performance metrics in Table 2 as the average MAE and predicted *R* ^2^ across all folds (from our 10‐folds cross validation) for each ROI in men and women, before application of the regression to the mean correction. Across all cardiac regions, age estimation models had greater error in men (higher MAE, lower *R* ^2^) than women. For both men and women, the greatest discrepancy between model estimated age and chronological age was observed for the RV followed by the myocardium, as indicated by greatest error in these models (higher MAE, lower *R* ^2^). In comparison, LV cavity age estimation models had the best performance metrics (lower MAE, higher *R* ^2^).

**TABLE 2:** Summary of Model Performance Metrics

Women					
	LV	RV	MYO	LA	RA
Mean MAE (years)	4.96	5.26	5.10	5.10	5.07
Mean predicted *R* ^2^	0.29	0.20	0.24	0.24	0.26
Correlation between predicted cardiac structure age and actual age	0.90	0.92	0.91	0.91	0.91

Men					
	LV	RV	MYO	LA	RA
Mean MAE (years)	5.33	5.49	5.42	5.36	5.37
Mean predicted *R* ^2^	0.22	0.17	0.19	0.21	0.20
Correlation between predicted cardiac structure age and actual age	0.92	0.93	0.93	0.92	0.92

Reported performance metrics are prior to application of the regression to the mean correction.

LA = left atrium; LV = left ventricle; MAE = mean absolute error; MYO = myocardium; RA = right atrium; RV = right ventricle.

### Left Atrium

**FIGURE 2:**
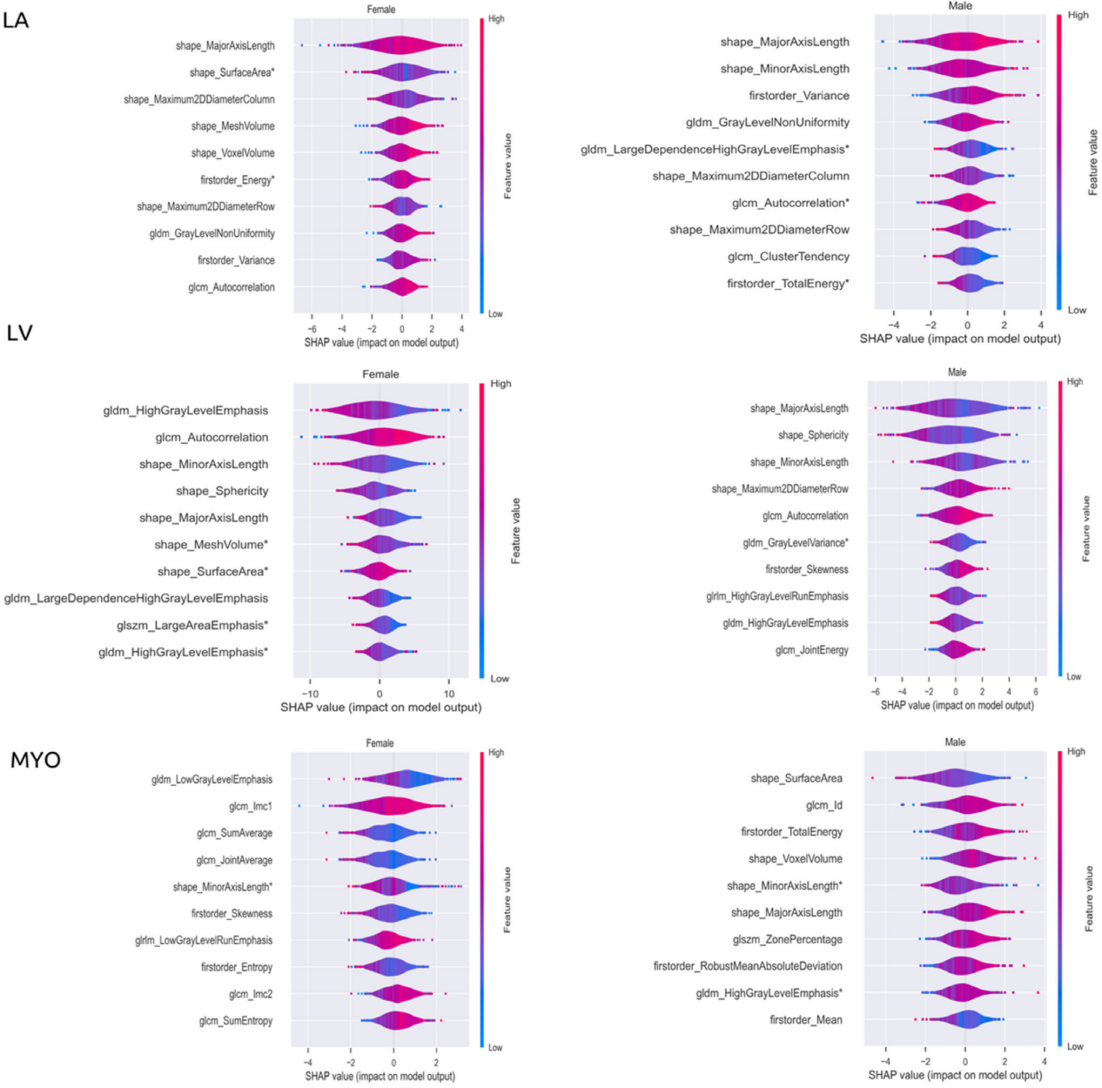
Top 10 more informative features for heart age models of left heart cardiac region for men and women, as identified by SHAP values. The x axis indicates the SHAP value range of each feature while the y axis displays the feature name. Each dot or circle in the plot indicates one subject in the model while the color shows how that feature is associated with the outcome. Red color indicates positive correlation while the blue color means negative correlation. A Zero SHAP value means the feature does not affect the outcome of the model. Asterisk indicates features extracted at end systole. SHAP = SHapley Additive exPlanations; GLCM = gray level co‐occurrence matrix; GLDM = gray‐level dependence matrix; GLRLM = gray‐level run length matrix; GLSZM = gray‐level size zone matrix.

Geometric alterations of the LA (radiomics shape features) were informative age‐related metrics (major and minor axis length, 2D diameter row, and column) in both women and men, all indicating that greater LA age was linked to smaller chamber size (Fig. 2). In women, we additionally observed that smaller surface area, mesh volume, and voxel volume were all linked to greater LA age. SI‐based features had a more minor role in these models, however overall, they indicated that greater LA age gap was linked to smoother less coarse texture in the LA blood pool (eg lower autocorrelation) in both men and women (Fig. 2).

### Left Ventricle

In both men and women, radiomics phenotypes indicating smaller and less spherical LV shape were among the most informative individual model features according to SHAP values (Fig. 2). Signal intensity (SI)‐based features also contributed importantly to models for men and women including features indicating greater skewness and variance in men. In women, features indicating greater autocorrelation of LV cavity pixel intensities (greater coarseness) and high gray‐level emphasis were informative. Another notable result is that the range of SHAP values in female cohorts was bigger than in the male cohort indicating greater impact of these features in the age model for women.

### LV Myocardium

In men, shape features were most informative to LV myocardial age estimation, while for women myocardial SI‐based features were more prominent (Fig. 2). In men, greater myocardial age was linked to smaller surface area, larger voxel volume, and smaller minor and major axis lengths. In women, myocardial age was indicated by features representing a dimmer and more homogenous pattern of myocardial SI. For instance, in women, greater myocardial age was linked to lower mean gray level intensity level (lower “joint average”), higher proportion of low SI pixel pairs in relation to high SI pairs (lower “sum average”), and less variation in intensity levels (lower “skewness,” higher “low run gray level emphasis”). The intensity variations related to myocardial age in men were in a similar direction to those in women but were less extensive and less informative to the overall model (Fig. 2).

### Right Atrium

**FIGURE 3:**
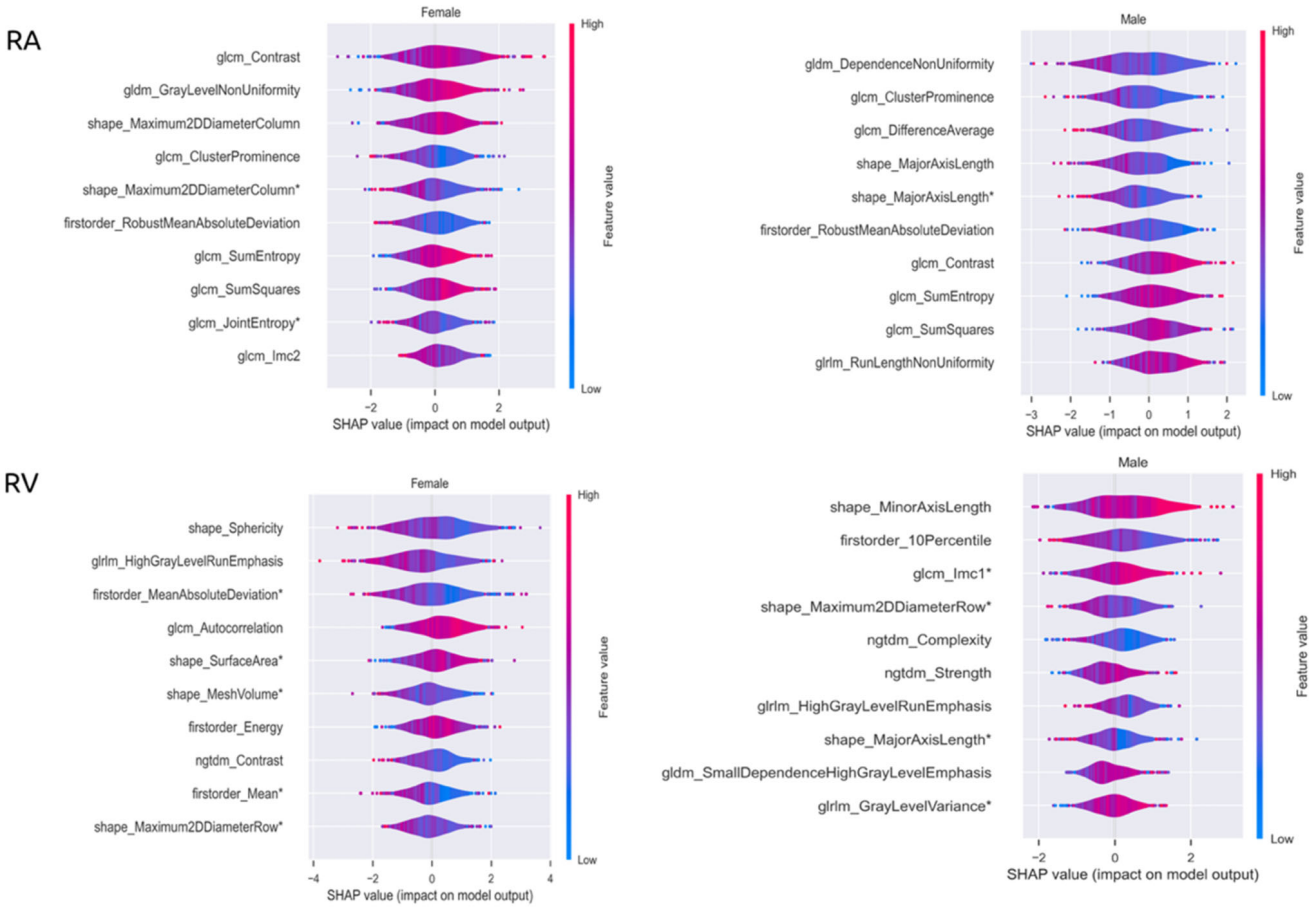
Top 10 more informative features for heart age models of right heart cardiac region for men and women, as identified by SHAP values. The x axis indicates the SHAP value range of each feature while the y axis displays the feature name. Each dot or circle in the plot indicates one subject in the model while the color shows how that feature is associated with the outcome. Red color indicates positive correlation while the blue color means negative correlation. A zero SHAP value means the feature does not affect the outcome of the model. Asterisk indicates features extracted at end systole. SHAP = SHapley Additive exPlanations; GLCM = gray‐level co‐occurrence matrix; GLDM = gray‐level dependence matrix; GLRLM = gray‐level run length matrix; GLSZM = gray‐level size zone matrix; NGTDM = neighboring gray tone difference matrix.

The list of most informative predictors produced by SHAP shows that the most informative features for the RA age model were dominated by texture features in both men and women (Fig. 3). In men, greater RA age was linked to smaller RA size (lower major axis length in end‐diastole and end‐systole), and greater homogeneity in RA (lower: “dependence nonuniformity,” “cluster prominence,” and “difference average”). In women, greater RA age was linked to larger RA size (higher “maximum 2D diameter column”) and higher heterogeneity of RA blood pool pixel intensities (higher: “gray level non‐uniformity,” “contrast,” “sum squares”).

### Right Ventricle

In women, greater RV age was linked to smaller chamber size (lower “mesh volume”, increasing R “maximum 2D diameter row”), less spherical RV shape (lower “sphericity”), and greater surface area of the cavity (higher “surface area”). In men, greater RV age was linked to larger “minor axis length,” lower “maximum 2D diameter,” and lower “major axis length” (Fig. 3). In women, increasing RV age was also linked to greater “autocorrelation,” indicating coarser pattern of blood pool SIs. In men, the informative SI‐based features indicated a less complex pattern of SIs (lower “complexity”) and greater variation in SI levels (“gray level variance”).

### Exposure Associations With Heart Age Gap

A total of 172 associations showed significant relationships with heart age gap in both women and men across the five ROIs. The largest number of associations was observed with the LV myocardium (*n* = 52) age gap with 52 significant associations divided into 27 in men and 25 in women. The LV (*n* = 44) and RV (*n* = 20) had the second and third highest number of significant associations with the tested exposures. On the other hand, LA had fewer associations with the tested exposures with only 15 significant associations, 8 in women and 7 in men (Table 3).

**TABLE 3:** The Number of Significant Associations in Each of the Five Regions Separated by Sex

Sex	LA	LV	MYO	RA	RV	Total
Men	7	23	27	9	17	83
Women	8	22	25	11	23	89
Total	15	45	52	20	40	172

LA = left atrium; LV = left ventricle; MYO = myocardium; RA = right atrium; RV = right ventricle.

**FIGURE 4:**
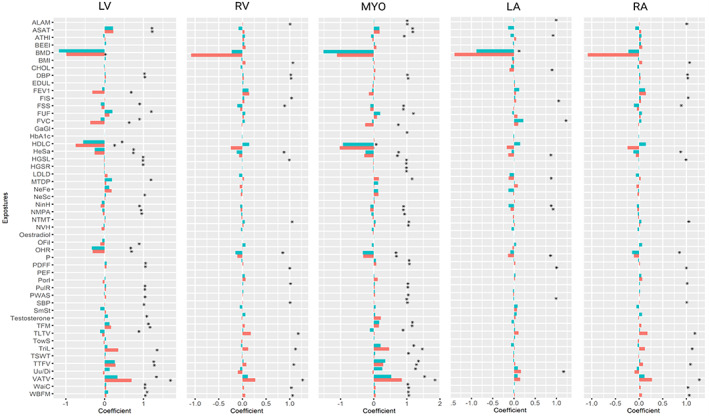
Association of all exposures with heart age gap in the five cardiac regions modeled for men and women. The x axis represents the coefficient value of the association of each exposure with the cardiac age gap while the y axis represents the exposure names. The coefficient values (beta) are not standardized which means every one unit increasing or decreasing in these exposures (independent variables) lead to increasing or decreasing in the heart age gap (dependent variable) in years based on the beta value when all other exposures are constant. Exposures with asterisk are statistically significant (corrected *P* value <0.05). Please refer to Table S3 Supplemental Material for full explanation of each exposure and its measurement units. BMD = heel bone mineral density; HDLC = HDL cholesterol; FVC = forced vital capacity; OHR = overall health rating; FEV1 = forced expiratory volume in 1‐second, best measure; HeSa = health satisfaction; ASAT = abdominal subcutaneous adipose tissue volume; TLTV = total lean tissue volume; NeFe = nervous feelings; NinH = number in household; LDLD = LDL direct; OFiI = oily fish intake; SmSt = smoking status; FSS = financial situation satisfaction; CHOL = cholesterol; Uu/Di = ever unenthusiastic/disinterested for a whole week; ATHI = average total household income before tax; NVH = number of vehicles in household; NMPA = number of days/week of moderate physical activity 10+ minutes; TriL = triglycerides level; TFM = trunk fat mass; MTDP = mouth/teeth dental problems; PorI = pork intake; BMI = body mass index; FUF = fed‐up feelings; EDUL = education level; TowS = Townsend score; PWAS = pulse wave arterial stiffness index (m/s); BEEI = beef_intake; FIS = fluid intelligence score; HGSL = hand grip strength (left); PDFF = 10P Liver PDFF (proton density fat fraction); TTFV = total trunk fat volume; TSWT = time spent watching television; NTMT = number of treatments/medications taken; HGSR = hand grip strength (right); HbA1c = glycated hemoglobin; WBFM = whole body fat mass; SBP = systolic blood pressure, automated reading (mmHg); ALAM = alanine aminotransferase; DBP = diastolic blood pressure, automated reading (mmHg); PulR = pulse rate; NeSc = neuroticism score; PEF = peak expiratory flow; GaGl = gamma glutamyltransferase; GaGl = gamma glutamyltransferase; WaiC = waist circumference; VATV = visceral adipose tissue volume.

In terms of the number of significant associations in each of the exposure categories, obesity and body composition metrics were dominant (51 significant associations), showing consistent associations between greater adiposity and with larger heart age gap across all cardiac structures (Table 4). Granular results of all exposure associations are available in Table S4 Supplemental Material and are summarized in Fig. 4.

**TABLE 4:** The Number of Significant Associations in Each of the Exposure Group Separated by Sex and Cardiac Structures

Exposures Groups	Sex	LA	LV	MYO	RA	RV	Total	
Blood biomarkers (*n* = 7)	Men	1	1	3	0	0	5	21
	Women	2	2	5	2	5	16	
Blood pressure and arterial stiffness (*n* = 4)	Men	1	2	3	2	2	10	24
	Women	0	4	4	2	4	14	
Lifestyle (*n* = 6)	Men	0	1	2	0	2	5	7
	Women	0	2	0	0	0	2	
Mental well‐being (*n* = 6)	Men	0	4	4	2	5	15	21
	Women	2	1	2	0	1	6	
Multiorgan health indicators (*n* = 10)	Men	3	6	5	5	1	20	40
	Women	3	5	6	1	5	20	
Obesity and body composition metrics (*n* = 9)	Men	0	8	8	0	7	23	51
	Women	0	7	7	6	8	28	
Socioeconomic (*n* = 5)	Men	2	0	2	0	0	4	7
	Women	1	1	1	0	0	3	
Sex hormones (*n* = 2)	Men	0	1	0	0	0	1	1
	Women	0	0	0	0	0	0	

LA = left atrium; LV = left ventricle; MYO = myocardium; RA = right atrium; RV = right ventricle.

Amongst the obesity measures considered, the strongest (largest magnitude) association was observed with visceral adiposity derived from abdominal MRI scans. Greater waist circumference was positively associated with heart age gap in both men and women. The relationships with obesity, across all the metrics, appeared stronger in women than in men. Higher high‐density lipoprotein (HDL) cholesterol was linked to greater heart age gap across the LV, RV, and myocardium, having stronger association in women than in men. The magnitude of this association appeared greatest with the LV myocardium age gap (higher HDL, smaller heart age gap). Higher levels of low‐density lipoprotein (LDL) cholesterol and triglyceride were linked to higher age gaps, although the magnitude of these associations was smaller than with HDL cholesterol.

Higher diastolic blood pressure, faster resting heart rate, and greater arterial stiffness were all significant positive associates of heart age gap, although the magnitude of these associations was small.

Indicators of better multiorgan health, such as higher hand grip strength (right, left), forced vital capacity, and heel bone mineral density were linked to smaller heart age gaps. Notably, for both men and women, better bone health as indicated by greater heel bone mineral density, showed the largest magnitude association with smaller heart age gap of all exposures considered.

In our sample, socioeconomic and lifestyle factors showed few significant associations with heart age gap. The number of vehicles in household, education level, and the Townsend score (measure of deprivation) did not show any significant (*P* value > 0.05) association with heart age gap. In terms of daily lifestyle factors, greater physical activity levels were linked to smaller heart age gap in men (myocardium, LV, RV) and women (LV). Smoking, beef, and pork intake did not show any significant (*P* value > 0.05) associations. Greater time spent watching television was associated with greater RV and myocardium heart age gaps in men. Higher testosterone level was observed with larger age LV age gap (greater biological aging) in men (coefficient 0.08, *P* value = 0.03).

## Discussion

In this study, we present age estimation models for key cardiac structures developed using cardiac MRI radiomics phenotypes in 18,117 UK Biobank participants free from clinical CVD. We selected this model due to its ability to handle the collinearity among the model predictors.^23^, ^24^ We considered discrepancy in age estimation from chronological age (heart age gap) as an indicator of greater cardiac aging, demonstrating differential aging patterns across heart structures and the associations of selected exposures with greater age gap.

Amongst the cardiac regions modeled, the LV age models had the best performance, while the RV models had the greatest error. This is in keeping with known greater anatomic complexity and irregularity of the RV^25^ compared to the LV, which is reflected in greater heterogeneity of RV phenotypes and greater error in our age models. Model performance showed greater error in men than women across all regions modeled, possibly indicating greater variation of IDPs in men.

The error in biological age estimation model comprises model error and biological age gap. In our study, we modeled biological age for the four cardiac chambers and the LV myocardium, observing different magnitude of error across these cardiac sites. This may reflect more advanced aging in cardiac regions with larger model error. For instance, the RV biological age estimation model had the largest MAE, which may indicate greater susceptibility of the RV to age‐related remodeling and greater biological aging in this chamber compared to other cardiac regions. The second largest error was in the model for myocardial biological age, which may highlight that the myocardium is also a site where age‐related alterations are prominent. In comparison, the LV had the smallest MAE of all chambers modeled, perhaps indicating that morphological age‐related alterations of the LV are less pronounced or occur at more advanced stages compared to other chambers. Alternatively, it is possible that the larger model error reflects “actual” error, that is, poorer model performance in age estimation for the RV and myocardium and better performance in age estimation for the LV. While it is not possible to definitively disentangle these two components of error, it is likely that they both contribute somewhat to the magnitude of MAE in our models.^26^

In evaluating the most informative features, overall, we observed importance of both shape and SI‐based radiomics features. Several features appeared informative across all ROI models. For instance, the major and minor axis length and surface area in the shape feature group were among the top informative predictors in the most regions and in both male and female cohorts. In addition, auto‐correlation from the texture feature group frequently appeared among the most informative features. The presence of these features in all examined regions in both male and female cohorts highlights their potential value as predictors in cardiac phenotype studies. The impact of the features on the model outcome was different from one ROI to another based on the SHAP value. For example, the range of the SHAP values for the features in the RA (female cohort) was smaller than in other regions. On the other hand, the impact of the features on the outcome was the largest when modeling LV age in women. Furthermore, the impact of the features on the outcome between male and female was different in some regions including LA, LV, and RV.

We evaluated associations of exposures with heart age gap metrics. The myocardial age gap had the largest number of significant associations, indicating that age‐related changes of the myocardium are importantly influenced by a wide range of different exposures. Obesity was a prominent associate of greater age gap across all cardiac structures, as represented by image‐derived measures of obesity, body size measures, and blood lipids. These associations were stronger in women than men. The myocardium and LV age gaps showed a greater number of significant associations with the exposures examined than the other regions, while LA and RA had fewer associations. In both men and women, significant associations between greater age gap of the LV, RV and myocardium were observed across a range of exposures including higher visceral adipose tissue volume, pulse rate, total trunk fat volume, abdominal subcutaneous adipose tissue volume, trunk fat mass, and whole bad fat mass. The most significant associations with myocardium and LV age gap were exposures from multiorgans indicators and obesity and body composition metrics.

MRI is unique as a modality in its ability to noninvasively characterize myocardial tissue. Previous work using MRI radiomics has demonstrated the value of radiomics SI‐based features extracted from the LV myocardium in discriminating disease states.^27^, ^28^, ^29^ The use of MRI radiomics is currently limited to research settings and further research is required before implementation in clinical settings. Other methods for myocardial tissue characterization include nonparametric mapping techniques and contrast‐enhanced image acquisitions. Late gadolinium enhancement (LGE) techniques are most established, and their clinical utility has been demonstrated in multiple previous studies in the setting of both ischemic and nonischemic cardiomyopathies.^30^, ^31^ The use of LGE acquisitions is accordingly widely adopted in clinical settings. Greater scan time (~15 minutes) and a small risk associated with intravenous gadolinium administration are drawbacks of this technique.^32^ Nonparametric mapping methods (T1, T2, T2*) have shown utility in disease discrimination and outcome prediction in multiple settings.^33^ These are noncontrast methods but do require dedicated specialist acquisitions. Although these methods are implemented in clinical practice, there are many outstanding technical issues, in particular regarding standardization of the techniques, that currently limited widespread generalizability.^34^ Furthermore, the role of these metrics in the setting of a healthy population is not yet definitively established.

Previous studies have examined associations of myocardial native T1 and T2 with increasing age. A large population study in the UK Biobank found increasing age‐related increase in myocardial native T1 in men and a decreasing trend in women.^35^ A smaller study of the Multi‐Ethnic Study of Atherosclerosis cohort reports positive association of native T1 with increasing age in men, but no significant age trend in women.^35^ The age dependency of T2 is less consistent, with some researchers reporting no relationship between T2 and age,^36^ while others report a decreasing trend.^37^ In our analysis, we used shape and SI‐based radiomics features extracted from bSSFPF short‐axis cine images. The association of these features with T1 and T2 extracted from mapping sequences is not known.

A key advantage of radiomics analysis is that it can be applied to existing standard of care contrast‐free images, presenting a potentially highly efficient method for tissue characterization. Our findings suggest that myocardial SI radiomics features may provide important information about myocardial aging in population cohorts. Our work encourages further research in this area to determine the clinical utility of this technique.

Of all the exposures considered, measures of obesity and serum lipids showed the most prominent associations with greater heart age gap across all the structures considered, appearing more important in women than men. Obesity is a global public health priority and its associations with adverse cardiovascular health are widely reported.^38^ Furthermore, others have reported phenotypic alterations of the LV in association with greater obesity^39^ The association of obesity exposures with greater heart age gap support the validity of our age estimation models. Furthermore, our study has described the associations of obesity with cardiac aging (as defined by age‐related phenotypic alterations) across all key cardiac chambers. Our observations highlight the importance of tackling obesity for alleviation of the global burden of CVD. The strongest associations were with abdominal MRI measures of obesity (visceral adipose tissue volume, abdominal subcutaneous adipose tissue volume, and total trunk fat volume). Notably, of the anthropometric measures of obesity, waist circumference showed stronger associations to larger heart age gap than body mass index, indicating the value of this metric in assessment of obesity‐related cardiovascular risk. Furthermore, although the baseline levels of obesity were greater for men, their associations with greater heart aging were stronger in women than men. This may indicate differential magnitude of the cardiovascular impact of obesity in women and warrants further dedicated study. Higher blood pressure, resting heart rate, and arterial stiffness were associated with significantly larger heart age gap across most cardiac regions. This observation highlights the utility of these established vascular health indicators as indices for monitoring heart aging.

### Limitations

The UK Biobank provided access to a large bank of uniformly acquired cardiac MR scans, which was essential for development of our models. The detailed characterization of participants permitted reliable ascertainment of health status using UK Biobank assessments and linked health records. However, given that our models were developed on a healthy population‐based cohort, further study is required to determine whether the observations made translate to a clinical cohort. Second, our models performed well within this dataset of homogenously acquired scans. However, cardiac MR radiomics features are susceptible to variation in pulse sequence parameters, scanner vendor, and case mix.^40^ Thus, these models may not perform in the same way in external cohorts. Third, our findings suggest that the age gap metrics extracted from our models may be useful as imaging biomarkers of cardiovascular health. However, direct comparison of our work with existing publications is challenging. Fourth, in this study, we used a simple model to estimate heart age to establish a benchmark for more complex methods with potentially better performance, as heart age modeling is not well established as it is for other organs such as brain age estimation. In the latter, there is a well‐described regression to the mean effect, which we also observed in our analysis. We opted to correct for this with the widely used method proposed by Beheshti et al.^22^ A limitation of this approach is false improvement of model performance^26^; with this in mind, we report precorrected performance metrics in this study. The correction is not expected to influence exposure associations with heart age gap (delta). Fifth, the clinical interpretation of the radiomics features in the model is not known as they have not been used and examined widely in morbidities and phenotypes to establish connections between the features and clinical outcomes. However, more work in this direction may solve the issues raised above. Further work is required to determine the clinical utility of these metrics. Moreover, we limit our study to participants without clinically diagnosed CVD. As we do not have access to clinically evaluate individual patients, we cannot exclude undiagnosed disease in the study sample.

### Conclusion

We demonstrate an interpretable model for biological age estimation across different cardiac structures developed using cardiac MRI radiomics phenotypes. Our findings indicate that discrepancy in image‐based age estimates and chronological age (heart age gap) may be a useful indicator of cardiovascular health and specifically for investigation of cardiovascular aging. A key advantage of the biological age estimation models presented in our study, is that the radiomics features extracted are obtained from routinely acquired standard of care cine MRI images. This means that our models have potential for broad application across research and clinical studies.

Our findings demonstrate obesity as an important correlate of heart aging, highlighting the importance of public health strategies for tackling obesity in ensuring population cardiovascular health. Further work is required to establish the potential wider utility of age gap metrics extracted from our models for risk estimation and outcome prediction.

## Author contributions

A.S. and Z.R.E. conceptualized the work and wrote the manuscript. E.R.P. and V.M.C. led on extraction and preparation of the radiomics features. A.S. led on all other analysis steps and prepared all figures and tables. T.E.N. provided expert critical review, and guidance on methods and interpretation of the results. C.M. supported the analysis. Z.R.E. and S.E.P provided overall supervision. N.C.H., S.N., and K.L. provided critical review of the work. All authors read and approved the final manuscript.

## Conflict of Interest

S.E.P. provides consultancy to Cardiovascular Imaging Inc, Calgary, Alberta, Canada. T.E.N. provides consultancy to Perspectum, Oxford, United Kingdom. The remaining authors have nothing to disclose.

## Ethics Statement

This study complies with the Declaration of Helsinki; the work was covered by the ethical approval for UK Biobank studies from the National Health Service (NHS) National Research Ethics Service on 17th June 2011 (Ref 11/NW/0382) and extended on 18 June 2021 (Ref 21/NW/0157) with written informed consent obtained from all participants.

## Consent for Publication

All the authors are accepted to publish the manuscript.

## Supporting information


**Data S1:** Supporting information

## Data Availability

The dataset supporting the conclusions of this article is available in the UK Biobank repository, under access application 2964, http://www.ukbiobank.ac.uk/register-apply. UK Biobank will make the data (including image acquisition parameters) available to all bona fide researchers for all types of health‐related research that is in the public interest, without preferential or exclusive access for any persons. All researchers will be subject to the same application process and approval criteria as specified by UK Biobank.
