# Supplementary material for: Image‐Based Biological Heart Age Estimation Reveals Differential Aging Patterns Across Cardiac Chambers
Source: J Magn Reson Imaging. 2023 Mar 16;58(6):1797–812. doi: 10.1002/jmri.28675 (PMC10947470; doi:10.1002/jmri.28675)
Supplement: Supplementary file 1 — Data S1: Supporting information [file JMRI-58-1797-s001.docx]

**SUPPLEMENTAL MATERIALS**

**Supplementary Table 1: Approach to ascertainment of cardiac diseases**

| Variable | UK Biobank field ID | Condition |
| --- | --- | --- |
| Self-report | 20002 | Angina |
|  |  | Heart attack/myocardial infarction |
|  |  | Mitral stenosis |
|  |  | Mitral valve disease |
|  |  | Heart valve problem/heart murmur |
|  |  | Mitral regurgitation / incompetence |
|  |  | Aortic valve disease |
|  |  | Aortic stenosis |
|  |  | Aortic regurgitation / incompetence |
|  |  | Cardiomyopathy |
|  |  | Hypertrophic cardiomyopathy (HCM / HOCM) |
|  |  | Heart failure/pulmonary odema |
|  |  | Sick sinus syndrome |
|  |  | Supraventricular tachycardia |
|  |  | Atrial fibrillation |
|  |  | Atrial flutter |
|  |  | Heart arrhythmia |
|  |  | Irregular heart beat |
| ICD-10 | 41202 | Angina pectoris |
|  |  | Other acute ischaemic heart diseases |
|  |  | Chronic ischaemic heart disease |
|  |  | Acute myocardial infarction |
|  |  | Subsequent myocardial infarction |
|  |  | Certain current complications following acute myocardial infarction |
|  |  | Mitral (valve) insufficiency |
|  |  | Nonrheumatic mitral (valve) stenosis |
|  |  | Other nonrheumatic mitral valve disorders |
|  |  | Non-rheumatic mitral valve disorder, unspecified |
|  |  | Non-rheumatic aortic valve disorders |
|  |  | Non-rheumatic tricuspid valve disorders |
|  |  | Pulmonary valve disorders |
|  |  | Endocarditis, valve unspecified |
|  |  | Mitral valve disorders in diseases classified elsewhere |
|  |  | Aortic valve disorders in diseases classified elsewhere |
|  |  | Pulmonary valve disorders in diseases classified elsewhere |
|  |  | Multiple valve disorders in diseases classified elsewhere |
|  |  | Endocarditis, valve unspecified, in diseases classified elsewhere |
|  |  | Cardiomyopathy |
|  |  | Cardiomyopathy in diseases classified elsewhere |
|  |  | Congestive heart failure |
|  |  | Left ventricular failure |
|  |  | Heart failure, unspecified |
|  |  | Atrioventricular block, second degree |
|  |  | Atrioventricular block, complete |
|  |  | Trifascicular block |
|  |  | Preexcitation syndrome |
|  |  | Cardiac arrest with successful resuscitation |
|  |  | Sudden cardiac death, so described |
|  |  | Cardiac arrest, unspecified |
|  |  | Re-entry ventricular arrhythmia |
|  |  | Supraventricular tachycardia |
|  |  | Ventricular tachycardia |
|  |  | Paroxysmal tachycardia, unspecified |
|  |  | Paroxysmal atrial fibrillation |
|  |  | Persistent atrial fibrillation |
|  |  | Chronic atrial fibrillation |
|  |  | Typical atrial flutter |
|  |  | Atypical atrial flutter |
|  |  | Atrial fibrillation and atrial flutter, unspecified |
|  |  | Ventricular fibrillation and flutter |
|  |  | Sick sinus syndrome |
|  |  | Rheumatic mitral valve diseases |
|  |  | Rheumatic aortic valve diseases |
|  |  | Rheumatic tricuspid valve diseases |
|  |  | Multiple valve diseases |
|  |  | Hypertensive heart disease |
|  |  | Hypertensive heart and renal disease |
| ICD-9 | 41203 | Coronary atherosclerosis |
|  |  | Other specified forms of chronic ischaemic heart disease |
|  |  | Chronic ischaemic heart disease, unspecified |
|  |  | Acute myocardial infarction |
|  |  | Other acute and subacute forms of ischaemic heart disease |
|  |  | Old myocardial infarction |
| Algorithm | 42000 | Date of myocardial infarction |
| Diagnosed by doctor | 6150 | Angina |
|  | 3627 | Age angina diagnosed |
|  | 6150 | Heart attack |
|  | 3894 | Age heart attack diagnosed |
| First occurrences | 131296 | Angina pectoris |
|  | 131304 | Other acute ischaemic heart diseases |
|  | 131306 | Chronic ischaemic heart disease |
|  | 131298 | Acute myocardial infarction |
|  | 131300 | Subsequent myocardial infarction |
|  | 131302 | Certain current complications following acute myocardial infarction |
|  | 131322 | Nonrheumatic mitral valve disorders |
|  | 131324 | Nonrheumatic aortic valve disorders |
|  | 131326 | Nonrheumatic tricuspid valve disorders |
|  | 131328 | Pulmonary valve disorders |
|  | 131330 | Endocarditis, valve unspecified |
|  | 131332 | Endocarditis and heart valve disorders in diseases classified elsewhere |
|  | 131338 | Cardiomyopathy |
|  | 131340 | Cardiomyopathy in diseases classified elsewhere |
|  | 131346 | Cardiac arrest |
|  | 131348 | Paroxysmal tachycardia |
|  | 131350 | Atrial fibrillation and flutter |
|  | 131276 | Rheumatic mitral valve diseases |
|  | 131278 | Rheumatic aortic valve diseases |
|  | 131280 | Rheumatic tricuspid valve diseases |
|  | 131282 | Multiple valve diseases |
|  | 131288 | Hypertensive heart disease |
|  | 131292 | Hypertensive heart and renal disease |
|  | 131354 | Heart failure |

**Supplementary Table 1 footnote.** ICD: international classification of disease.

**Supplementary Table 2. List of radiomic features used to estimate cardiac age**

| shape_Elongation | glcm_SumEntropy |
| --- | --- |
| shape_MajorAxisLength | glcm_SumSquares |
| shape_Maximum2DDiameterColumn | gldm_DependenceEntropy |
| shape_Maximum2DDiameterRow | gldm_DependenceNonUniformity |
| shape_Maximum2DDiameterSlice | gldm_DependenceNonUniformityNormalized |
| shape_Maximum3DDiameter | gldm_DependenceVariance |
| shape_MeshVolume | gldm_GrayLevelNonUniformity |
| shape_MinorAxisLength | gldm_GrayLevelVariance |
| shape_Sphericity | gldm_HighGrayLevelEmphasis |
| shape_SurfaceArea | gldm_LargeDependenceEmphasis |
| shape_SurfaceVolumeRatio | gldm_LargeDependenceHighGrayLevelEmphasis |
| shape_VoxelVolume | gldm_LargeDependenceLowGrayLevelEmphasis |
| firstorder_10Percentile | gldm_LowGrayLevelEmphasis |
| firstorder_90Percentile | gldm_SmallDependenceEmphasis |
| firstorder_Energy | gldm_SmallDependenceHighGrayLevelEmphasis |
| firstorder_Entropy | gldm_SmallDependenceLowGrayLevelEmphasis |
| firstorder_InterquartileRange | glrlm_GrayLevelNonUniformity |
| firstorder_Kurtosis | glrlm_GrayLevelNonUniformityNormalized |
| firstorder_Maximum | glrlm_GrayLevelVariance |
| firstorder_MeanAbsoluteDeviation | glrlm_HighGrayLevelRunEmphasis |
| firstorder_Mean | glrlm_LongRunEmphasis |
| firstorder_Median | glrlm_LongRunHighGrayLevelEmphasis |
| firstorder_Minimum | glrlm_LongRunLowGrayLevelEmphasis |
| firstorder_Range | glrlm_LowGrayLevelRunEmphasis |
| firstorder_RobustMeanAbsoluteDeviation | glrlm_RunEntropy |
| firstorder_RootMeanSquared | glrlm_RunLengthNonUniformity |
| firstorder_Skewness | glrlm_RunLengthNonUniformityNormalized |
| firstorder_TotalEnergy | glrlm_RunPercentage |
| firstorder_Uniformity | glrlm_RunVariance |
| firstorder_Variance | glrlm_ShortRunEmphasis |
| glcm_Autocorrelation | glrlm_ShortRunHighGrayLevelEmphasis |
| glcm_ClusterProminence | glrlm_ShortRunLowGrayLevelEmphasis |
| glcm_ClusterShade | glszm_GrayLevelNonUniformity |
| glcm_ClusterTendency | glszm_GrayLevelNonUniformityNormalized |
| glcm_Contrast | glszm_GrayLevelVariance |
| glcm_Correlation | glszm_HighGrayLevelZoneEmphasis |
| glcm_DifferenceAverage | glszm_LargeAreaEmphasis |
| glcm_DifferenceEntropy | glszm_LargeAreaHighGrayLevelEmphasis |
| glcm_DifferenceVariance | glszm_LargeAreaLowGrayLevelEmphasis |
| glcm_Id | glszm_LowGrayLevelZoneEmphasis |
| glcm_Idm | glszm_SizeZoneNonUniformity |
| glcm_Idmn | glszm_SizeZoneNonUniformityNormalized |
| glcm_Idn | glszm_SmallAreaEmphasis |
| glcm_Imc1 | glszm_SmallAreaHighGrayLevelEmphasis |
| glcm_Imc2 | glszm_SmallAreaLowGrayLevelEmphasis |
| glcm_InverseVariance | glszm_ZoneEntropy |
| glcm_JointAverage | glszm_ZonePercentage |
| glcm_JointEnergy | glszm_ZoneVariance |
| glcm_JointEntropy | ngtdm_Busyness |
| glcm_MCC | ngtdm_Coarseness |
| glcm_MaximumProbability | ngtdm_Complexity |
| glcm_SumAverage | ngtdm_Contrast |
|  | ngtdm_Strength |

**Supplementary Table 2 footnote.** Each feature was extracted twice (end-systole, end-diastole) from each cardiac region resulting in a total of 210 features for each region. GLCM: gray-level co-occurrence matrix; GLRLM: gray-level run-length matrix; GLSZM: gray-level size-zone matrix; NGTDM: neighboring gray tone difference matrix; GLDM: gray-level dependence matrix.

**Supplementary Table 3: Number of features and subjects included in modelling for each region of interest after feature selection and outlier removal procedures**

| Women | | | | | |
| --- | --- | --- | --- | --- | --- |
|  | LV | RV | MYO | LA | RA |
| Original number of features | 210 | | | | |
| Optimal number of features using RFECV | 125 | 91 | 121 | 102 | 65 |
| Original number of subjects | 15095 | | | | |
| Number of subjects free of heart diseases | 9779 | | | | |
| Final number of subjects after applying outliers’ removal | 9402 | 9402 | 9416 | 9419 | 9409 |
| Men | | | | | |
|  | LV | RV | MYO | LA | RA |
| Original number of features | 210 | | | | |
| Optimal number of features using RFECV | 118 | 95 | 43 | 91 | 58 |
| Original number of subjects | 14049 | | | | |
| Number of subjects free of heart diseases | 8338 | | | | |
| Final number of subjects after applying outliers’ removal | 8040 | 8051 | 8049 | 8065 | 8070 |

**Supplementary Table 3 footnote**. LA: left atrium; LV: left ventricle; MYO: myocardium; RA: right atrium; RV: right ventricle; RFECV: recursive feature elimination with cross-validation.

**Supplementary Table 4. Associations of all exposures with heart age gap from the five cardiac regions modelled**

| Coefficient | Sex | Anatomy | P-value | Exposures |
| --- | --- | --- | --- | --- |
| -1.51788 | Male | MYO | 7.44E-06 | Heel bone mineral density (BMD) |
| -1.39966 | Female | LA | 5.29E-04 | Heel bone mineral density (BMD) |
| -1.17606 | Male | LV | 2.26E-03 | Heel bone mineral density (BMD) |
| -1.11439 | Female | MYO | 3.14E-03 | Heel bone mineral density (BMD) |
| -1.08438 | Female | RA | 2.37E-02 | Heel bone mineral density (BMD) |
| -1.02737 | Female | MYO | 7.71E-21 | HDL cholesterol |
| -0.98516 | Female | LV | 1.66E-02 | Heel bone mineral density (BMD) |
| -0.92931 | Male | MYO | 3.55E-13 | HDL cholesterol |
| -0.88149 | Male | LA | 2.85E-02 | Heel bone mineral density (BMD) |
| -0.74638 | Female | RV | 4.35E-02 | Heel bone mineral density (BMD) |
| -0.74552 | Female | LV | 1.65E-10 | HDL cholesterol |
| -0.66669 | Male | RV | 8.73E-02 | Heel bone mineral density (BMD) |
| -0.55235 | Male | LV | 1.80E-04 | HDL cholesterol |
| -0.52283 | Female | RV | 6.23E-07 | HDL cholesterol |
| -0.37027 | Female | LV | 1.62E-07 | Forced vital capacity (FVC) |
| -0.33161 | Male | LV | 3.53E-09 | Overall health rating |
| -0.33055 | Male | MYO | 2.88E-10 | Overall health rating |
| -0.31878 | Female | MYO | 1.40E-09 | Overall health rating |
| -0.31826 | Female | LV | 1.44E-03 | Forced expiratory volume in 1-second (FEV1), Best measure |
| -0.31065 | Female | LV | 3.72E-08 | Overall health rating |
| -0.31039 | Male | RV | 6.88E-02 | HDL cholesterol |
| -0.28894 | Female | MYO | 1.93E-11 | Health satisfaction |
| -0.26208 | Female | LV | 1.73E-08 | Health satisfaction |
| -0.26087 | Female | MYO | 2.03E-04 | Forced vital capacity (FVC) |
| -0.25658 | Male | LV | 1.57E-07 | Health satisfaction |
| -0.25254 | Male | MYO | 3.76E-08 | Health satisfaction |
| -0.24338 | Female | RA | 5.91E-02 | HDL cholesterol |
| -0.23725 | Male | RV | 7.66E-06 | Overall health rating |
| -0.23346 | Female | RV | 4.98E-06 | Overall health rating |
| -0.22582 | Male | RA | 5.47E-01 | Heel bone mineral density (BMD) |
| -0.21006 | Female | RV | 2.13E-02 | Forced expiratory volume in 1-second (FEV1), Best measure |
| -0.17409 | Male | RV | 1.81E-04 | Health satisfaction |
| -0.17015 | Female | LA | 1.99E-01 | HDL cholesterol |
| -0.14991 | Female | MYO | 1.15E-01 | Forced expiratory volume in 1-second (FEV1), Best measure |
| -0.14897 | Female | RV | 7.75E-04 | Health satisfaction |
| -0.14705 | Male | RA | 1.51E-02 | Overall health rating |
| -0.14154 | Female | LA | 1.54E-02 | Overall health rating |
| -0.13961 | Male | LA | 1.93E-01 | Abdominal subcutaneous adipose tissue volume |
| -0.13593 | Male | RV | 8.29E-05 | Total lean tissue volume |
| -0.13376 | Female | LA | 8.60E-03 | Health satisfaction |
| -0.1296 | Male | LA | 4.60E-01 | Nervous feelings |
| -0.12773 | Female | RV | 5.17E-02 | Forced vital capacity (FVC) |
| -0.12686 | Male | LA | 3.61E-04 | Number in household |
| -0.12681 | Female | LA | 1.63E-02 | LDL direct |
| -0.12084 | Male | RA | 2.28E-02 | Health satisfaction |
| -0.11941 | Male | LV | 1.34E-03 | Total lean tissue volume |
| -0.11917 | Male | MYO | 7.04E-04 | Total lean tissue volume |
| -0.11265 | Female | LV | 1.22E-02 | Oily fish intake |
| -0.11152 | Male | LV | 8.68E-02 | Smoking status |
| -0.10913 | Female | MYO | 3.08E-03 | Number in household |
| -0.1088 | Female | MYO | 1.11E-02 | Financial situation satisfaction |
| -0.1084 | Male | RA | 2.28E-02 | Financial situation satisfaction |
| -0.10796 | Female | RA | 1.14E-01 | Overall health rating |
| -0.10623 | Female | LA | 1.57E-02 | Cholesterol |
| -0.10468 | Female | LV | 8.63E-03 | Number in household |
| -0.10392 | Male | LA | 7.75E-02 | LDL direct |
| -0.10277 | Male | LV | 2.62E-02 | Forced vital capacity (FVC) |
| -0.10262 | Male | MYO | 3.14E-03 | Number in household |
| -0.10195 | Male | MYO | 9.93E-03 | Financial situation satisfaction |
| -0.09994 | Male | LV | 1.87E-02 | Financial situation satisfaction |
| -0.09562 | Female | RA | 4.58E-01 | Ever unenthusiastic/disinterested for a whole week |
| -0.095 | Male | RV | 1.04E-02 | Financial situation satisfaction |
| -0.08802 | Female | RV | 3.31E-02 | Total lean tissue volume |
| -0.08728 | Male | LA | 1.42E-01 | Overall health rating |
| -0.08236 | Female | LV | 7.50E-02 | Financial situation satisfaction |
| -0.08224 | Male | LA | 3.18E-02 | Average total household income before tax |
| -0.08182 | Female | LA | 4.83E-02 | Number in household |
| -0.08132 | Female | LV | 9.87E-02 | Number of vehicles in household |
| -0.08122 | Male | RA | 8.15E-01 | Abdominal subcutaneous adipose tissue volume |
| -0.08046 | Male | MYO | 2.39E-02 | Average total household income before tax |
| -0.07284 | Male | LV | 5.79E-02 | Number in household |
| -0.07158 | Male | MYO | 5.04E-06 | Number of days/week of moderate physical activity 10+ minutes |
| -0.06936 | Female | RA | 4.58E-01 | Health satisfaction |
| -0.06776 | Female | RV | 7.69E-02 | Oily fish intake |
| -0.06762 | Male | RV | 1.38E-01 | Number of vehicles in household |
| -0.0672 | Male | RA | 4.08E-01 | LDL direct |
| -0.06705 | Male | LA | 9.87E-02 | Triglycerides Level |
| -0.06692 | Female | LV | 1.65E-01 | Total lean tissue volume |
| -0.06683 | Male | LA | 9.59E-02 | Cholesterol |
| -0.06426 | Male | LV | 3.54E-01 | Forced expiratory volume in 1-second (FEV1), Best measure |
| -0.06389 | Male | LV | 1.48E-01 | Oily fish intake |
| -0.06373 | Male | RV | 7.11E-02 | Number in household |
| -0.06278 | Male | LV | 2.87E-04 | Number of days/week of moderate physical activity 10+ minutes |
| -0.0592 | Male | MYO | 2.21E-01 | Number of vehicles in household |
| -0.05888 | Male | LA | 3.81E-01 | Trunk fat mass |
| -0.05866 | Male | LA | 2.16E-01 | Number of vehicles in household |
| -0.05742 | Male | MYO | 2.30E-01 | Oily fish intake |
| -0.05595 | Male | LA | 4.60E-01 | Health satisfaction |
| -0.05535 | Female | RA | 5.95E-01 | Mouth/teeth dental problems |
| -0.05496 | Male | RA | 4.23E-01 | Smoking status |
| -0.05114 | Male | MYO | 2.51E-01 | Forced vital capacity (FVC) |
| -0.05054 | Male | MYO | 4.02E-01 | Forced expiratory volume in 1-second (FEV1), Best measure |
| -0.04976 | Male | RA | 7.64E-01 | Ever unenthusiastic/disinterested for a whole week |
| -0.0492 | Female | LV | 4.87E-01 | Pork intake |
| -0.04887 | Male | RV | 3.04E-03 | Number of days/week of moderate physical activity 10+ minutes |
| -0.04792 | Female | LV | 9.75E-03 | Number of days/week of moderate physical activity 10+ minutes |
| -0.04781 | Female | RV | 2.51E-01 | Financial situation satisfaction |
| -0.047 | Male | RA | 3.34E-01 | Number in household |
| -0.04639 | Male | LA | 3.98E-01 | Body mass index |
| -0.04586 | Female | RA | 4.58E-01 | Financial situation satisfaction |
| -0.04369 | Male | LA | 8.32E-01 | Fed-up feelings |
| -0.0418 | Female | LV | 5.58E-01 | Ever unenthusiastic/disinterested for a whole week |
| -0.04022 | Male | RV | 6.48E-01 | Smoking status |
| -0.04 | Female | MYO | 3.36E-01 | Average total household income before tax |
| -0.03887 | Female | MYO | 5.66E-01 | Ever unenthusiastic/disinterested for a whole week |
| -0.03859 | Female | LA | 4.50E-01 | Oily fish intake |
| -0.03811 | Male | LV | 3.25E-01 | Average total household income before tax |
| -0.03676 | Female | RA | 4.63E-01 | Number in household |
| -0.03588 | Male | RA | 4.08E-01 | Cholesterol |
| -0.03545 | Female | MYO | 9.43E-02 | Education level |
| -0.03415 | Female | RA | 8.01E-01 | Nervous feelings |
| -0.03325 | Female | RV | 8.81E-02 | Education level |
| -0.03246 | Male | RA | 4.33E-01 | Triglycerides Level |
| -0.03099 | Male | RA | 5.78E-01 | Number of vehicles in household |
| -0.03061 | Female | MYO | 7.75E-02 | Number of days/week of moderate physical activity 10+ minutes |
| -0.02994 | Female | LA | 4.71E-01 | Body mass index |
| -0.02902 | Male | LV | 5.02E-01 | Number of vehicles in household |
| -0.02847 | Female | LV | 7.95E-02 | Townsend score |
| -0.02833 | Male | LA | 2.11E-01 | Pulse wave Arterial Stiffness index (m/s) |
| -0.02726 | Male | RV | 3.63E-01 | Average total household income before tax |
| -0.02699 | Female | LA | 1.85E-01 | Number of days/week of moderate physical activity 10+ minutes |
| -0.02687 | Female | RV | 8.81E-02 | Townsend score |
| -0.02619 | Male | MYO | 4.27E-01 | Body mass index |
| -0.02575 | Male | LA | 6.58E-01 | Beef intake |
| -0.02514 | Female | MYO | 6.75E-01 | Number of vehicles in household |
| -0.02496 | Male | RA | 2.97E-01 | Number of days/week of moderate physical activity 10+ minutes |
| -0.02457 | Female | RA | 2.82E-01 | Townsend score |
| -0.02456 | Male | RV | 6.48E-01 | Oily fish intake |
| -0.0241 | Male | RV | 6.48E-01 | Beef intake |
| -0.02117 | Male | RV | 2.26E-01 | Fluid intelligence score |
| -0.02081 | Female | MYO | 3.44E-04 | Hand grip strength (left) |
| -0.02042 | Male | MYO | 2.21E-01 | Education level |
| -0.02037 | Female | LA | 2.64E-01 | Pulse wave Arterial Stiffness index (m/s) |
| -0.02021 | Male | RA | 8.15E-01 | Liver PDFF (proton density fat fraction) |
| -0.01972 | Male | RA | 9.29E-01 | Nervous feelings |
| -0.01845 | Female | RV | 7.43E-01 | Number in household |
| -0.01814 | Female | RA | 3.27E-01 | Number of days/week of moderate physical activity 10+ minutes |
| -0.01775 | Male | LA | 6.64E-01 | Total trunk fat volume |
| -0.01773 | Female | LA | 1.85E-01 | Time spent watching television |
| -0.01771 | Male | LA | 3.90E-01 | Number of treatments/medications taken |
| -0.01744 | Male | LV | 8.41E-01 | Cholesterol |
| -0.01714 | Male | RA | 8.51E-01 | Body mass index |
| -0.01705 | Male | MYO | 7.01E-01 | Cholesterol |
| -0.01653 | Female | LA | 8.32E-01 | Nervous feelings |
| -0.01607 | Male | MYO | 9.93E-05 | Hand grip strength (right) |
| -0.01597 | Male | LV | 4.33E-01 | Pulse wave Arterial Stiffness index (m/s) |
| -0.01587 | Male | MYO | 3.41E-01 | Fluid intelligence score |
| -0.01524 | Female | RV | 7.69E-03 | Hand grip strength (left) |
| -0.01502 | Female | RV | 7.69E-03 | Hand grip strength (right) |
| -0.014996 | Female | RA | 8.80E-01 | Testosterone |
| -0.01498 | Male | RV | 3.63E-01 | Education level |
| -0.01495 | Female | LV | 1.66E-02 | Hand grip strength (left) |
| -0.01481 | Female | RA | 8.21E-01 | Forced vital capacity (FVC) |
| -0.01445 | Female | MYO | 9.89E-03 | Hand grip strength (right) |
| -0.01441 | Female | LA | 8.32E-01 | Financial situation satisfaction |
| -0.01416 | Male | MYO | 7.38E-04 | Hand grip strength (left) |
| -0.01402 | Female | LV | 9.85E-01 | Testosterone |
| -0.0139 | Male | LA | 8.43E-02 | Glycated haemoglobin (HbA1c) |
| -0.01383 | Male | LA | 4.20E-01 | Time spent watching television |
| -0.01332 | Female | RV | 3.88E-01 | Number of days/week of moderate physical activity 10+ minutes |
| -0.01329 | Female | RA | 3.27E-01 | Time spent watching television |
| -0.01174 | Male | LV | 8.41E-01 | LDL direct |
| -0.01157 | Male | LV | 1.63E-02 | Hand grip strength (left) |
| -0.01151 | Male | LA | 6.64E-01 | Whole body fat mass |
| -0.01122 | Male | LA | 2.26E-07 | Systolic blood pressure, automated reading (mmHg) |
| -0.01112 | Male | LV | 1.63E-02 | Hand grip strength (right) |
| -0.01095 | Female | LV | 8.07E-02 | Hand grip strength (right) |
| -0.01008 | Female | MYO | 8.36E-01 | Oily fish intake |
| -0.01006 | Female | LA | 5.31E-01 | Townsend score |
| -0.00996 | Female | LV | 5.78E-01 | Education level |
| -0.00976 | Male | RA | 4.08E-01 | Glycated haemoglobin (HbA1c) |
| -0.00938 | Male | LA | 1.07E-03 | Alanine aminotransferase |
| -0.00935 | Male | RA | 4.38E-02 | Hand grip strength (left) |
| -0.00922 | Female | LA | 3.30E-01 | Glycated haemoglobin (HbA1c) |
| -0.00864 | Female | RV | 2.36E-01 | Glycated haemoglobin (HbA1c) |
| -0.00798 | Male | RV | 8.73E-02 | Hand grip strength (left) |
| -0.00797 | Male | LA | 6.64E-01 | Liver PDFF (proton density fat fraction) |
| -0.00778 | Female | LA | 8.37E-01 | Beef intake |
| -0.00764 | Male | MYO | 1.20E-04 | Systolic blood pressure, automated reading (mmHg) |
| -0.00706 | Male | RA | 4.92E-01 | Time spent watching television |
| -0.00659 | Male | RV | 9.75E-01 | Cholesterol |
| -0.00619 | Male | RV | 1.91E-01 | Hand grip strength (right) |
| -0.00618 | Male | RA | 1.76E-01 | Hand grip strength (right) |
| -0.0061 | Male | RA | 9.31E-01 | Mouth/teeth dental problems |
| -0.00603 | Female | RA | 9.15E-01 | Smoking status |
| -0.0058 | Male | RV | 7.53E-01 | Pulse wave Arterial Stiffness index (m/s) |
| -0.00558 | Male | LA | 2.00E-01 | Diastolic blood pressure, automated reading (mmHg) |
| -0.00541 | Female | LA | 9.66E-01 | Abdominal subcutaneous adipose tissue volume |
| -0.00486 | Male | LA | 7.52E-01 | Number of days/week of moderate physical activity 10+ minutes |
| -0.00475 | Female | LA | 1.99E-01 | Alanine aminotransferase |
| -0.00418 | Male | RV | 6.83E-01 | Glycated haemoglobin (HbA1c) |
| -0.00408 | Male | LV | 6.67E-02 | Systolic blood pressure, automated reading (mmHg) |
| -0.00401 | Female | LA | 2.64E-01 | Pulse rate |
| -0.00371 | Male | MYO | 7.01E-01 | Glycated haemoglobin (HbA1c) |
| -0.00358 | Male | LA | 8.49E-01 | Neuroticism score |
| -0.00352 | Female | RA | 6.52E-01 | Hand grip strength (left) |
| -0.00341 | Female | LV | 9.05E-01 | Body mass index |
| -0.00339 | Female | RA | 7.75E-01 | Glycated haemoglobin (HbA1c) |
| -0.00316 | Female | LA | 1.62E-01 | Systolic blood pressure, automated reading (mmHg) |
| -0.00231 | Female | RA | 7.42E-01 | Hand grip strength (right) |
| -0.00186 | Female | RA | 9.64E-01 | Number of vehicles in household |
| -0.00175 | Female | RA | 8.78E-01 | Neuroticism score |
| -0.00081 | Female | LV | 8.07E-02 | Peak expiratory flow (PEF) |
| -0.0008 | Male | RA | 7.40E-01 | Alanine aminotransferase |
| -0.00072 | Male | LA | 4.58E-01 | Gamma glutamyltransferase |
| -0.00059 | Male | RA | 7.72E-01 | Systolic blood pressure, automated reading (mmHg) |
| -0.00047 | Female | RV | 9.85E-01 | Body mass index |
| -0.00046 | Male | RV | 8.82E-01 | Oestradiol |
| -0.0003 | Female | RV | 5.25E-01 | Peak expiratory flow (PEF) |
| -0.00027 | Female | MYO | 6.35E-01 | Peak expiratory flow (PEF) |
| -0.00015 | Female | RV | 9.91E-01 | Number of treatments/medications taken |
| -0.00013 | Female | LA | 8.19E-01 | Peak expiratory flow (PEF) |
| -9.15E-05 | Male | RV | 9.98E-01 | LDL direct |
| -6.88E-05 | Female | MYO | 6.04E-01 | Oestradiol |
| -4.08E-05 | Female | RA | 8.80E-01 | Oestradiol |
| -3.48E-05 | Male | RV | 8.93E-01 | Peak expiratory flow (PEF) |
| -3.27E-06 | Female | LV | 9.85E-01 | Oestradiol |
| 2.17E-05 | Female | LA | 8.69E-01 | Oestradiol |
| 2.76E-05 | Female | RV | 8.20E-01 | Oestradiol |
| 0.000118 | Female | LV | 9.24E-01 | Gamma glutamyltransferase |
| 0.000323 | Female | LA | 9.51E-01 | Hand grip strength (right) |
| 0.000354 | Male | LV | 2.69E-01 | Peak expiratory flow (PEF) |
| 0.000406 | Female | LA | 9.66E-01 | Waist circumference |
| 0.000409 | Male | MYO | 1.83E-01 | Peak expiratory flow (PEF) |
| 0.000425 | Female | LA | 7.49E-01 | Gamma glutamyltransferase |
| 0.000732 | Female | RA | 1.88E-01 | Peak expiratory flow (PEF) |
| 0.000859 | Female | MYO | 9.46E-01 | Townsend score |
| 0.000874 | Male | LA | 1.15E-02 | Peak expiratory flow (PEF) |
| 0.000923 | Male | RA | 9.76E-03 | Peak expiratory flow (PEF) |
| 0.000964 | Female | RA | 6.40E-01 | Gamma glutamyltransferase |
| 0.001009 | Male | RV | 7.53E-01 | Systolic blood pressure, automated reading (mmHg) |
| 0.001025 | Male | RA | 9.29E-01 | Neuroticism score |
| 0.001036 | Male | RA | 4.08E-01 | Gamma glutamyltransferase |
| 0.00104 | Male | RV | 9.74E-01 | Body mass index |
| 0.00114 | Female | MYO | 9.46E-01 | Fluid intelligence score |
| 0.001144 | Male | MYO | 5.07E-01 | Oestradiol |
| 0.001147 | Male | RV | 3.37E-01 | Gamma glutamyltransferase |
| 0.001317 | Male | LV | 8.41E-01 | Glycated haemoglobin (HbA1c) |
| 0.001333 | Male | LV | 3.87E-01 | Gamma glutamyltransferase |
| 0.001419 | Female | LV | 9.72E-01 | Beef_intake |
| 0.001551 | Male | MYO | 1.48E-01 | Gamma glutamyltransferase |
| 0.00162 | Female | LA | 9.68E-01 | Number of vehicles in household |
| 0.00212 | Male | LA | 6.70E-01 | Hand grip strength (right) |
| 0.002178 | Male | LV | 6.62E-01 | Alanine aminotransferase |
| 0.002202 | Male | RA | 9.54E-01 | Trunk fat mass |
| 0.002232 | Male | LV | 8.92E-01 | Fluid intelligence score |
| 0.002275 | Female | LV | 9.24E-01 | Glycated haemoglobin (HbA1c) |
| 0.002476 | Male | LV | 3.27E-01 | Oestradiol |
| 0.002503 | Female | RV | 2.76E-02 | Gamma glutamyltransferase |
| 0.002796 | Female | LA | 7.54E-01 | Hand grip strength (left) |
| 0.003098 | Male | RV | 3.37E-01 | Alanine aminotransferase |
| 0.003178 | Female | LV | 5.08E-01 | Alanine aminotransferase |
| 0.003229 | Female | MYO | 7.22E-03 | Gamma glutamyltransferase |
| 0.0036 | Male | LA | 7.93E-02 | Oestradiol |
| 0.003685 | Female | RV | 9.23E-01 | Number of vehicles in household |
| 0.003753 | Male | RA | 8.19E-02 | Oestradiol |
| 0.003826 | Male | LA | 2.11E-01 | Pulse rate |
| 0.003916 | Male | RA | 9.40E-01 | Whole body fat mass |
| 0.004289 | Male | RV | 8.82E-01 | Testosterone |
| 0.004591 | Female | RA | 2.03E-01 | Pulse rate |
| 0.004768 | Male | RA | 7.10E-01 | Townsend score |
| 0.005053 | Male | MYO | 9.06E-01 | LDL direct |
| 0.00509 | Female | RA | 9.15E-01 | Oily fish intake |
| 0.005167 | Male | RA | 8.51E-01 | Waist circumference |
| 0.006194 | Female | RV | 2.76E-02 | Alanine aminotransferase |
| 0.006251 | Female | RA | 8.59E-01 | Cholesterol |
| 0.006333 | Female | RV | 7.60E-01 | Fluid intelligence score |
| 0.006958 | Female | RA | 8.60E-01 | Education level |
| 0.007081 | Male | MYO | 4.98E-03 | Alanine aminotransferase |
| 0.007166 | Female | LA | 9.76E-02 | Diastolic blood pressure, automated reading (mmHg) |
| 0.007544 | Male | LA | 8.49E-01 | Financial situation satisfaction |
| 0.008225 | Female | RA | 3.35E-02 | Alanine aminotransferase |
| 0.008231 | Male | LA | 1.12E-01 | Hand grip strength (left) |
| 0.008304 | Female | RA | 1.45E-05 | Systolic blood pressure, automated reading (mmHg) |
| 0.008628 | Female | LV | 9.24E-01 | Cholesterol |
| 0.008963 | Female | RV | 1.57E-07 | Systolic blood pressure, automated reading (mmHg) |
| 0.009011 | Female | MYO | 3.00E-03 | Alanine aminotransferase |
| 0.010129 | Female | RA | 5.89E-01 | Pulse wave Arterial Stiffness index (m/s) |
| 0.010259 | Female | LV | 1.04E-07 | Systolic blood pressure, automated reading (mmHg) |
| 0.01159 | Female | MYO | 8.36E-01 | Smoking status |
| 0.011616 | Female | LV | 5.57E-01 | Fluid intelligence score |
| 0.012859 | Male | LA | 8.59E-01 | Mouth/teeth dental problems |
| 0.013018 | Female | LV | 4.80E-01 | Number of treatments/medications taken |
| 0.013066 | Female | RV | 2.51E-01 | Neuroticism score |
| 0.013227 | Female | LV | 3.14E-01 | Neuroticism score |
| 0.013374 | Female | LA | 8.94E-01 | Trunk fat mass |
| 0.013573 | Male | RV | 4.96E-01 | Number of treatments/medications taken |
| 0.013734 | Female | MYO | 9.30E-01 | Mouth/teeth dental problems |
| 0.013779 | Male | MYO | 3.32E-05 | Diastolic blood pressure, automated reading (mmHg) |
| 0.013796 | Female | MYO | 3.84E-14 | Systolic blood pressure, automated reading (mmHg) |
| 0.013914 | Male | LA | 4.30E-01 | Waist circumference |
| 0.014259 | Male | MYO | 7.62E-01 | Pork intake |
| 0.014809 | Female | LV | 2.77E-01 | Time spent watching television |
| 0.015045 | Female | MYO | 6.11E-02 | Glycated haemoglobin (HbA1c) |
| 0.015446 | Female | RV | 7.43E-01 | Average total household income before tax |
| 0.015643 | Female | LA | 7.49E-01 | Triglycerides Level |
| 0.016005 | Male | MYO | 2.21E-01 | Townsend score |
| 0.016016 | Male | RA | 2.46E-07 | Pulse rate |
| 0.016899 | Male | RA | 8.51E-01 | Total lean tissue volume |
| 0.017276 | Male | RV | 5.33E-08 | Diastolic blood pressure, automated reading (mmHg) |
| 0.017366 | Female | LA | 2.39E-01 | Neuroticism score |
| 0.017673 | Female | MYO | 9.40E-02 | Time spent watching television |
| 0.017893 | Male | RA | 4.94E-01 | Pulse wave Arterial Stiffness index (m/s) |
| 0.017952 | Female | RV | 1.80E-09 | Pulse rate |
| 0.018164 | Male | MYO | 1.21E-01 | Neuroticism score |
| 0.018332 | Female | LV | 5.78E-01 | Average total household income before tax |
| 0.01842 | Male | LV | 7.14E-01 | Pork intake |
| 0.018513 | Female | RA | 3.39E-01 | Liver PDFF (proton density fat fraction) |
| 0.018655 | Female | LA | 4.71E-01 | Whole body fat mass |
| 0.018734 | Female | MYO | 5.49E-01 | Body mass index |
| 0.019297 | Male | RV | 8.25E-01 | Forced expiratory volume in 1-second (FEV1), Best measure |
| 0.02002 | Male | LV | 3.25E-01 | Education level |
| 0.020101 | Male | LA | 2.56E-01 | Education level |
| 0.020173 | Female | RV | 3.81E-11 | Diastolic blood pressure, automated reading (mmHg) |
| 0.020384 | Male | RA | 4.00E-01 | Education level |
| 0.02047 | Female | LA | 3.76E-01 | Education level |
| 0.020695 | Male | RV | 6.49E-01 | Pork intake |
| 0.02131 | Male | RV | 1.31E-13 | Pulse rate |
| 0.021573 | Female | RV | 7.69E-02 | Time spent watching television |
| 0.021827 | Male | LV | 2.69E-01 | Number of treatments/medications taken |
| 0.022173 | Male | LV | 1.68E-10 | Diastolic blood pressure, automated reading (mmHg) |
| 0.022195 | Female | LA | 2.36E-01 | Number of treatments/medications taken |
| 0.022448 | Female | MYO | 6.07E-01 | Total lean tissue volume |
| 0.02258 | Male | RA | 6.87E-11 | Diastolic blood pressure, automated reading (mmHg) |
| 0.022957 | Female | RA | 1.88E-01 | Number of treatments/medications taken |
| 0.023372 | Male | RA | 8.51E-01 | Total trunk fat volume |
| 0.023783 | Male | LA | 6.64E-01 | Total lean tissue volume |
| 0.024256 | Male | RV | 1.78E-01 | Liver PDFF (proton density fat fraction) |
| 0.02427 | Male | LV | 6.07E-02 | Time spent watching television |
| 0.024882 | Female | MYO | 5.11E-02 | Neuroticism score |
| 0.025958 | Female | MYO | 2.62E-16 | Pulse rate |
| 0.025995 | Male | LA | 7.74E-02 | Townsend score |
| 0.02676 | Male | MYO | 2.33E-02 | Waist circumference |
| 0.02712 | Female | LA | 6.78E-01 | Pork intake |
| 0.028351 | Male | RV | 7.11E-02 | Townsend score |
| 0.028363 | Male | RV | 1.04E-02 | Neuroticism score |
| 0.028678 | Male | MYO | 5.77E-01 | Beef intake |
| 0.028733 | Female | RV | 2.35E-03 | Waist circumference |
| 0.029013 | Female | LA | 6.47E-01 | Total trunk fat volume |
| 0.029054 | Male | MYO | 1.64E-23 | Pulse rate |
| 0.029402 | Male | LA | 1.24E-01 | Fluid intelligence score |
| 0.029977 | Female | RA | 8.01E-01 | Fed-up feelings |
| 0.030005 | Male | LV | 3.88E-01 | Body mass index |
| 0.030019 | Male | MYO | 1.14E-01 | Pulse wave Arterial Stiffness index (m/s) |
| 0.030209 | Male | RV | 4.61E-03 | Time spent watching television |
| 0.030333 | Female | RA | 4.26E-20 | Diastolic blood pressure, automated reading (mmHg) |
| 0.030973 | Male | LV | 5.79E-02 | Townsend score |
| 0.031016 | Male | LV | 1.70E-02 | Neuroticism score |
| 0.032119 | Female | LA | 2.04E-01 | Liver PDFF (proton density fat fraction) |
| 0.032471 | Female | LV | 1.81E-22 | Pulse rate |
| 0.032482 | Female | LV | 6.96E-01 | Smoking status |
| 0.03291 | Female | LV | 1.81E-22 | Diastolic blood pressure, automated reading (mmHg) |
| 0.033713 | Male | RV | 3.38E-03 | Waist circumference |
| 0.03381 | Female | RA | 1.34E-03 | Waist circumference |
| 0.034311 | Female | RA | 6.40E-01 | LDL direct |
| 0.034434 | Male | LA | 6.58E-01 | Pork intake |
| 0.034603 | Female | RA | 4.63E-01 | Average total household income before tax |
| 0.03513 | Female | LV | 1.05E-03 | Waist circumference |
| 0.035547 | Female | MYO | 1.32E-28 | Diastolic blood pressure, automated reading (mmHg) |
| 0.03557 | Male | LV | 4.92E-01 | Beef intake |
| 0.036067 | Male | LV | 2.63E-31 | Pulse rate |
| 0.037388 | Female | RV | 1.54E-02 | Whole body fat mass |
| 0.037587 | Female | RV | 2.84E-02 | Pulse wave Arterial Stiffness index (m/s) |
| 0.03759 | Male | RA | 4.38E-02 | Fluid intelligence score |
| 0.038001 | Female | LV | 4.97E-02 | Pulse wave Arterial Stiffness index (m/s) |
| 0.03804 | Female | LA | 8.69E-01 | Testosterone |
| 0.038597 | Male | LV | 1.67E-03 | Waist circumference |
| 0.038675 | Female | MYO | 3.56E-02 | Pulse wave Arterial Stiffness index (m/s) |
| 0.03923 | Male | RA | 4.23E-01 | Beef intake |
| 0.040347 | Male | MYO | 2.87E-02 | Whole body fat mass |
| 0.040519 | Female | RV | 8.20E-01 | Testosterone |
| 0.040767 | Female | RA | 8.31E-02 | Fluid intelligence score |
| 0.041228 | Female | LV | 5.57E-01 | Mouth/teeth dental problems |
| 0.042962 | Female | MYO | 4.47E-03 | Number of treatments/medications taken |
| 0.04317 | Male | RV | 1.94E-02 | Whole body fat mass |
| 0.044335 | Female | RV | 3.95E-01 | Smoking status |
| 0.044336 | Male | MYO | 2.81E-01 | Testosterone |
| 0.044729 | Male | MYO | 1.95E-05 | Time spent watching television |
| 0.044991 | Female | RV | 1.14E-02 | Liver PDFF (proton density fat fraction) |
| 0.045188 | Female | RA | 3.65E-01 | Abdominal subcutaneous adipose tissue volume |
| 0.045695 | Female | LV | 1.03E-02 | Whole body fat mass |
| 0.047249 | Female | LA | 7.54E-01 | Forced expiratory volume in 1-second (FEV1), Best measure |
| 0.047391 | Female | LV | 1.71E-02 | Liver PDFF (proton density fat fraction) |
| 0.04784 | Female | RV | 4.49E-01 | Ever unenthusiastic/disinterested for a whole week |
| 0.048272 | Male | LA | 4.20E-01 | Oily fish intake |
| 0.049317 | Female | MYO | 1.58E-01 | Cholesterol |
| 0.050001 | Male | RA | 4.23E-01 | Pork intake |
| 0.050022 | Female | LA | 1.33E-02 | Fluid intelligence score |
| 0.050123 | Male | RA | 2.87E-01 | Forced vital capacity (FVC) |
| 0.05041 | Female | RA | 1.94E-01 | Trunk fat mass |
| 0.050594 | Female | RV | 1.38E-01 | Cholesterol |
| 0.050966 | Male | RA | 1.51E-02 | Number of treatments/medications taken |
| 0.051335 | Female | LA | 2.50E-01 | Average total household income before tax |
| 0.051703 | Male | LV | 6.29E-03 | Liver PDFF (proton density fat fraction) |
| 0.051759 | Female | MYO | 2.10E-07 | Waist circumference |
| 0.051997 | Male | RA | 3.34E-01 | Average total household income before tax |
| 0.055749 | Male | MYO | 4.24E-01 | Smoking status |
| 0.057337 | Female | MYO | 4.63E-04 | Whole body fat mass |
| 0.057784 | Male | MYO | 9.18E-04 | Number of treatments/medications taken |
| 0.0595 | Female | RA | 1.06E-03 | Whole body fat mass |
| 0.060208 | Male | RA | 7.64E-01 | Fed-up feelings |
| 0.061018 | Male | RV | 1.91E-01 | Forced vital capacity (FVC) |
| 0.06288 | Male | LA | 7.93E-02 | Testosterone |
| 0.063131 | Male | RA | 8.19E-02 | Testosterone |
| 0.065033 | Male | RA | 2.97E-01 | Oily fish intake |
| 0.066165 | Male | MYO | 3.72E-04 | Liver PDFF (proton density fat fraction) |
| 0.066824 | Female | RA | 2.58E-02 | Body mass index |
| 0.067624 | Female | RA | 3.27E-01 | Beef intake |
| 0.068038 | Female | RA | 3.27E-01 | Pork intake |
| 0.069743 | Male | LV | 2.11E-01 | Triglycerides Level |
| 0.071108 | Female | LA | 4.05E-01 | Smoking status |
| 0.073117 | Female | MYO | 8.24E-05 | Liver PDFF (proton density fat fraction) |
| 0.0748 | Male | RV | 9.63E-02 | Triglycerides Level |
| 0.076552 | Female | LV | 2.46E-01 | LDL direct |
| 0.078694 | Female | MYO | 7.75E-02 | Beef intake |
| 0.078778 | Female | RV | 7.69E-02 | Beef intake |
| 0.080425 | Female | LA | 3.61E-01 | Fed-up feelings |
| 0.08075 | Male | LV | 3.11E-02 | Testosterone |
| 0.082287 | Female | RV | 2.51E-01 | Fed-up feelings |
| 0.08569 | Female | RA | 3.29E-02 | Total trunk fat volume |
| 0.085981 | Male | LA | 3.81E-01 | Visceral adipose tissue volume |
| 0.086189 | Female | RV | 7.69E-02 | Pork intake |
| 0.086298 | Male | LA | 4.20E-01 | Smoking status |
| 0.090747 | Female | LA | 2.89E-01 | Mouth/teeth dental problems |
| 0.091294 | Male | LA | 4.60E-01 | Ever unenthusiastic/disinterested for a whole week |
| 0.091561 | Male | LV | 5.23E-06 | Whole body fat mass |
| 0.095059 | Female | RV | 1.80E-01 | Mouth/teeth dental problems |
| 0.09871 | Female | MYO | 1.79E-01 | Fed-up feelings |
| 0.099598 | Female | LA | 2.36E-01 | Forced vital capacity (FVC) |
| 0.100327 | Female | RV | 2.51E-01 | Nervous feelings |
| 0.108701 | Female | LA | 1.09E-01 | Total lean tissue volume |
| 0.114163 | Female | RV | 1.47E-02 | LDL direct |
| 0.114343 | Male | RA | 3.71E-01 | Visceral adipose tissue volume |
| 0.115573 | Male | LV | 2.05E-01 | Nervous feelings |
| 0.116879 | Female | MYO | 7.75E-02 | Pork intake |
| 0.120035 | Female | LV | 1.44E-01 | Fed-up feelings |
| 0.122298 | Female | RA | 4.65E-02 | Triglycerides Level |
| 0.122706 | Male | LA | 1.12E-01 | Forced expiratory volume in 1-second (FEV1), Best measure |
| 0.124078 | Male | LV | 1.67E-03 | Trunk fat mass |
| 0.127514 | Male | RA | 7.19E-02 | Forced expiratory volume in 1-second (FEV1), Best measure |
| 0.128558 | Male | LV | 1.13E-01 | Ever unenthusiastic/disinterested for a whole week |
| 0.12921 | Male | RV | 6.33E-02 | Ever unenthusiastic/disinterested for a whole week |
| 0.132066 | Male | MYO | 1.22E-01 | Nervous feelings |
| 0.133002 | Male | MYO | 8.46E-02 | Mouth/teeth dental problems |
| 0.140762 | Female | MYO | 1.05E-01 | Nervous feelings |
| 0.141867 | Male | RV | 8.73E-02 | Mouth/teeth dental problems |
| 0.142461 | Male | RA | 4.08E-01 | HDL cholesterol |
| 0.144279 | Female | RA | 1.88E-01 | Forced expiratory volume in 1-second (FEV1), Best measure |
| 0.145804 | Female | LA | 2.04E-01 | Visceral adipose tissue volume |
| 0.146015 | Male | LA | 3.14E-01 | HDL cholesterol |
| 0.153246 | Female | MYO | 1.59E-03 | LDL direct |
| 0.154065 | Female | MYO | 1.87E-05 | Trunk fat mass |
| 0.164722 | Female | LA | 4.52E-02 | Ever unenthusiastic/disinterested for a whole week |
| 0.165059 | Male | MYO | 1.86E-05 | Trunk fat mass |
| 0.165441 | Female | LV | 2.17E-05 | Trunk fat mass |
| 0.166173 | Male | RV | 8.94E-06 | Trunk fat mass |
| 0.170246 | Male | MYO | 5.88E-03 | Abdominal subcutaneous adipose tissue volume |
| 0.175765 | Female | LV | 7.50E-02 | Nervous feelings |
| 0.176136 | Female | MYO | 4.63E-04 | Abdominal subcutaneous adipose tissue volume |
| 0.179568 | Female | RA | 3.91E-04 | Total lean tissue volume |
| 0.188082 | Female | RV | 1.50E-08 | Trunk fat mass |
| 0.190211 | Male | LV | 1.71E-02 | Mouth/teeth dental problems |
| 0.190316 | Male | RV | 1.57E-03 | Abdominal subcutaneous adipose tissue volume |
| 0.194299 | Male | MYO | 9.93E-03 | Fed-up feelings |
| 0.200027 | Male | LV | 1.70E-02 | Fed-up feelings |
| 0.204291 | Male | MYO | 1.31E-08 | Triglycerides Level |
| 0.210139 | Male | RV | 1.62E-07 | Total trunk fat volume |
| 0.213111 | Female | MYO | 6.38E-02 | Testosterone |
| 0.21504 | Male | LV | 1.12E-03 | Abdominal subcutaneous adipose tissue volume |
| 0.221961 | Male | LA | 1.81E-06 | Forced vital capacity (FVC) |
| 0.222448 | Female | LV | 4.44E-05 | Abdominal subcutaneous adipose tissue volume |
| 0.233626 | Male | RV | 9.34E-03 | Nervous feelings |
| 0.233738 | Female | RV | 7.56E-07 | Abdominal subcutaneous adipose tissue volume |
| 0.246222 | Male | RV | 7.91E-04 | Fed-up feelings |
| 0.253944 | Male | RV | 5.86E-06 | Visceral adipose tissue volume |
| 0.256255 | Female | RV | 3.21E-13 | Total trunk fat volume |
| 0.258549 | Male | MYO | 1.04E-03 | Ever unenthusiastic/disinterested for a whole week |
| 0.26172 | Male | LV | 9.68E-10 | Total trunk fat volume |
| 0.26459 | Female | RV | 3.30E-08 | Triglycerides Level |
| 0.273218 | Female | RA | 1.34E-03 | Visceral adipose tissue volume |
| 0.276088 | Female | LV | 6.48E-12 | Total trunk fat volume |
| 0.285929 | Female | MYO | 4.05E-14 | Total trunk fat volume |
| 0.32992 | Male | LV | 2.92E-08 | Visceral adipose tissue volume |
| 0.347695 | Female | LV | 5.85E-11 | Triglycerides Level |
| 0.347953 | Male | MYO | 2.65E-19 | Total trunk fat volume |
| 0.462521 | Female | MYO | 7.71E-21 | Triglycerides Level |
| 0.53184 | Male | MYO | 5.39E-23 | Visceral adipose tissue volume |
| 0.564127 | Female | RV | 8.34E-14 | Visceral adipose tissue volume |
| 0.691806 | Female | LV | 9.78E-16 | Visceral adipose tissue volume |
| 0.846548 | Female | MYO | 1.69E-26 | Visceral adipose tissue volume |

**Supplementary Table 4 footnote**. HDL: high-density lipoprotein; LA: left atrium; LDL: low-density lipoprotein; LV: left ventricle; MYO: myocardium; RA: right atrium; RV: right ventricle.
